# Fully Wireless and Flexible Valves for Multiplexed and Prolonged Intravesical Liquid Release

**DOI:** 10.1002/adhm.71197

**Published:** 2026-05-07

**Authors:** Boyang Xiao, Yi Zhu, Yusheng Wang, Janene M. Pierce, Jeffrey J. Tosoian, Xiaoguang Dong

**Affiliations:** ^1^ Department of Mechanical Engineering Vanderbilt University Nashville Tennessee USA; ^2^ Department of Urology Vanderbilt University Medical Center Nashville Tennessee USA; ^3^ Vanderbilt‐Ingram Cancer Center Nashville Tennessee USA; ^4^ Department of Biomedical Engineering Vanderbilt University Nashville Tennessee USA; ^5^ Vanderbilt Institute for Surgery and Engineering Vanderbilt University Nashville Tennessee USA

**Keywords:** implantable device, magnetic valve, multiplexed, soft robotics, wirelessly controlled drug delivery

## Abstract

Minimally invasive, long‐term, and precisely controlled drug delivery is essential for treating bladder diseases such as interstitial cystitis and bladder cancer. However, conventional approaches, including injection‐based delivery and indwelling catheters, offer limited controllability, cause patient discomfort, and increase the risk of infection and tissue irritation. Existing intravesical devices further lack active control over drug release, are restricted to single therapeutic agents, and may induce bladder overactivity due to continuous mechanical stimulation. Here, we present a strategy to remotely control multiple flexible magnetic valves on a soft robotic patch for controlled, multiplexed, and sustained liquid delivery. The device integrates magnetic valves with soft osmotic pumps to achieve precise dosing, selective release, and on‐demand mixing of multiple therapeutics. Release rates are tuned by modulating valve duty cycles, while coordinated multi‐valve actuation enables independent ejection and programmable mixing. A bioadhesive soft patch provides stable attachment to wet bladder tissue for over seven days. Wireless, selective valve control is achieved using a portable magnetic actuation system with wireless sensing feedback. Phantom and ex vivo porcine bladder studies demonstrate robust adhesion, controlled multiplexed delivery, and long‐term operational stability. This platform establishes a foundation for minimally invasive and on‐demand intravesical therapy for precision medicine.

## Graphical abstract

A fundamental mechanism for the simultaneous control of multiple magnetic valves is reported in a miniature soft robotic patch for intravesical drug delivery, enabling fully wireless and programmable liquid release. Integrated with a bioadhesive interface and induction‐coupling‐based liquid volume sensing, the soft robotic patch is promising to enable long‐term retention and prolonged, closed‐loop therapy in patients with bladder diseases.

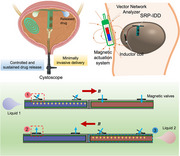

## 1 Introduction

Sustained drug release is essential for managing bladder diseases such as bladder cancer and interstitial cystitis (IC), which profoundly affect urinary function and quality of life [1]. Bladder cancer, characterized by abnormal or malignant cell proliferation, can be classified as non‐muscle‐invasive or muscle‐invasive and is often associated with hematuria, frequent urination, and pelvic pain [2]. Its management typically requires continuous intravesical chemotherapy. In contrast, IC, also known as bladder pain syndrome, is a chronic noninfectious condition marked by pelvic pain, urinary urgency, and frequency without an identifiable cause [3, 4]. Among available treatments, intravesical drug delivery [5, 6] offers a minimally invasive route that administers therapeutics directly into the bladder, minimizing systemic side effects and improving drug bioavailability compared to oral or systemic administration.

However, existing intravesical drug delivery methods remain limited. The most common approach is short‐term instillation via urinary catheters, but it suffers from low drug retention due to irritative symptoms and urine dilution. Although advanced methods such as electromotive drug administration (EMDA) [7, 8, 9] enhance tissue penetration by applying electrical currents, they still provide only transient therapeutic effects and require frequent re‐instillations for prolonged efficacy [10]. Recent local drug delivery systems, such as microrobotic systems [11, 12, 13, 14] and nano/micro‐drug release system incorporated with mucoadhesive hydrogels [15, 16, 17, 18], offer precise, targeted drug or gene delivery, but maintaining effective local drug concentrations over extended durations longer than a week remains a challenge [19]. Sustained‐release systems, such as device‐based approaches like TAR‐200 [21, 22, 23], improve treatment duration by remaining in the bladder for extended periods. Nevertheless, these devices typically lack active control over the release rate, are often limited to a single therapeutic agent, and can cause discomfort or increased urinary frequency due to mechanical interaction with bladder tissues, particularly at the bladder base.

There remains an unmet need for intravesical drug delivery devices capable of achieving prolonged, controllable release while ensuring reliable retention and minimizing patient discomfort. Existing electrically actuated pumps [24, 25, 26, 27] typically rely on batteries or wireless charging, which limit operational duration and actuation range. Osmotic pressure–driven pumps [28, 29, 30], though energy‐efficient, lack precise control over the rate, timing, and sequence of drug release. In contrast, magnetically actuated valves [31, 32, 33, 34] offer a promising route for fully wireless operation, enabling remote control with tunable release dynamics, extended actuation distance, and programmable delivery sequences. However, coordinating multiple magnetic valves within an implantable platform remains a major challenge due to the magnetic coupling among the valves, which hinders independent and multiplexed regulation.

To overcome these limitations, we introduce a strategy for the simultaneous control of multiple magnetic valves and demonstrate its implementation in a soft robotic patch for intravesical drug delivery (SRP‐IDD). The integrated magnetic valves regulate soft osmotic pumps to enable precise, on‐demand dosing. We first establish controlled valve actuation to regulate liquid release rates, followed by demonstrating multiplexed liquid ejection that allows the independent release of two distinct drugs or on‐demand mixing and co‐release of therapeutic combinations. Real‐time resonance‐frequency monitoring provides quantitative feedback on liquid release, ensuring accurate and programmable dosing. In addition, a bioadhesive layer enables stable attachment to bladder tissues for more than seven days, effectively reducing patient discomfort by minimizing contact with the bladder base. The SRP‐IDD can be intravesically delivered via a cystoscope and wirelessly controlled using a portable magnetic actuation unit. With its integrated functionalities, especially the coordinated programmable magnetic valves, the reported intravesical drug delivery system enables active, programmable control over the dosage and timing of releasing multiple drugs, which is difficult to achieve in existing drug delivery platforms. Collectively, this magnetic‐valve‐enabled soft robotic system represents a versatile, long‐term therapeutic platform for wireless, minimally invasive, and precise treatment of bladder diseases.

## 2 Results

### 2.1 Overall Concept of the SRP‐IDD for Controlled Liquid Release

**FIGURE 1:**
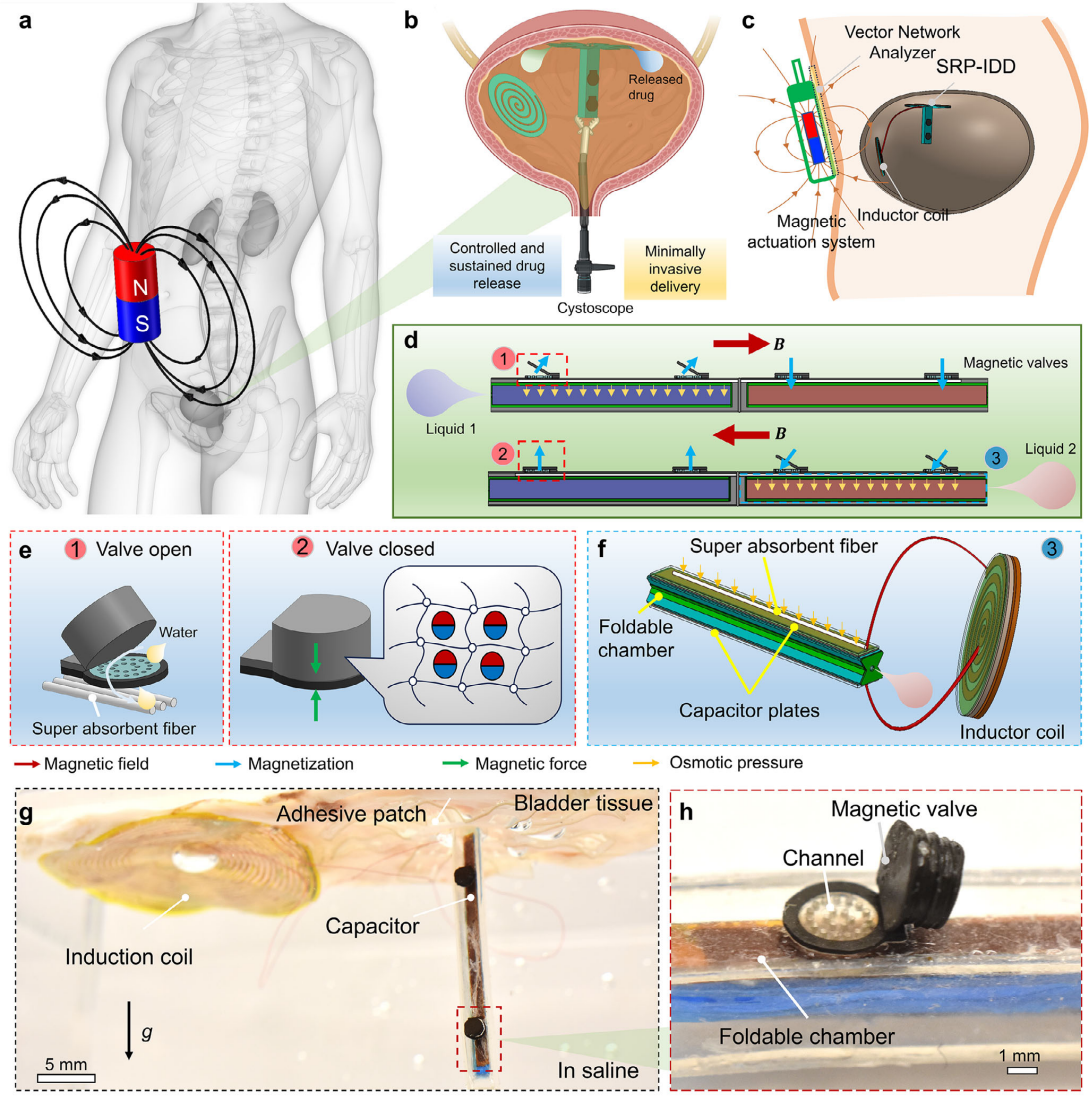
Overview of the concept and design of the magnetic valve‐enabled SRP‐IDD. (a) Illustration of the magnetic valve‐enabled soft robotic device in the bladder. (b) Illustration of the device delivered and anchored onto the bladder tissue surface. (c) Illustration of the device positioned inside the bladder, integrated with the portable magnetic actuation system and signal pickup system. (d) Schematic of coordinated multiple magnetic valve control for liquid release. The valves are actuated by an external magnetic field to open, allowing superabsorbent fibers (SAF) to drive the foldable chambers and expel different liquids. (e) Illustration of the valve opening and closing by applying an external magnetic field. (f) Illustration of the pumping mechanism by a foldable chamber and swelling SAF with an LC circuit for sensing liquid release volume. (g) Optical image of the device deployed on porcine bladder tissue in saline, showing the drug release unit with valves, pumps, a capacitor‐based volume sensing module, and an adhesive patch. (h) Zoom‐in view of the magnetic valve and foldable drug chamber, highlighting valve control of pump operation.

The SRP‐IDD consists of a drug release pump with multiple flexible magnetic valves, a soft adhesive patch, and an induction coil for wireless sensing and communication (Figure 1a). It is delivered inside a bladder via a cystoscope as illustrated in Figure 1b. The outer diameter of the SRP‐IDD is designed to be 3.5 mm after folding to allow its delivery by a cystoscope. After being deployed in a bladder, the SRP‐IDD can stay in urine and release the drug on demand. Figure 1c illustrates the actuation of the SRP‐IDD by a portable magnetic actuation system. A wireless pickup coil and its signal processing unit allow monitoring of the drug release volume of the device.

A key feature of the device is the integration of multiple magnetic valves that coordinately regulate both the pumping rate and multiplexed liquid release (Figure 1d). Pumping is driven by the swelling of a superabsorbent fiber (SAF), which absorbs ambient water through the inlet and expands to deform the foldable chamber laterally. This deformation ejects the loaded drugs through the outlet. Each magnetic soft valve enables remote, selective control of liquid release (Figure 1e). When an external magnetic field is applied parallel to the magnetic leaf, the generated torque overcomes the magnetic attraction, opening the valve at approximately 10 mT. Another essential feature is an inductor‐capacitor (LC) circuit attached to the foldable chamber, where the capacitance of the parallel plate capacitor on the foldable chamber varies with the chamber deformation (Figure 1f). This capacitance change shifts the resonant frequency of the LC circuit, which can be wirelessly detected by an external pickup coil via radio‐frequency (RF) communication and mapped to the liquid volume inside the chamber.

As a proof‐of‐concept demonstration, Figure 1g shows the SRP‐IDD attached to a porcine bladder tissue sample and submerged in saline solution (0.9 wt.% NaCl). The SRP‐IDD consists of a drug chamber integrated with magnetic valves and a capacitive sensing element, an adhesive patch for long‐term retention, and an induction coil for wireless signal transmission. For implantation inside the bladder, stable and reliable underwater tissue adhesion is essential. The adhesive patch incorporates a dry hydrogel adhesive that enables firm attachment to the bladder tissue surface for several days, even under continuous liquid immersion. The magnetic valves integrated along the outer shell are highlighted in Figure 1h. At rest, the magnetic leaf and base attract each other due to their predefined magnetization profiles, effectively preventing accidental valve activation.

### 2.2 Mechanism of Controlling Liquid Delivery by Magnetically Actuated Flexible Valves

**FIGURE 2:**
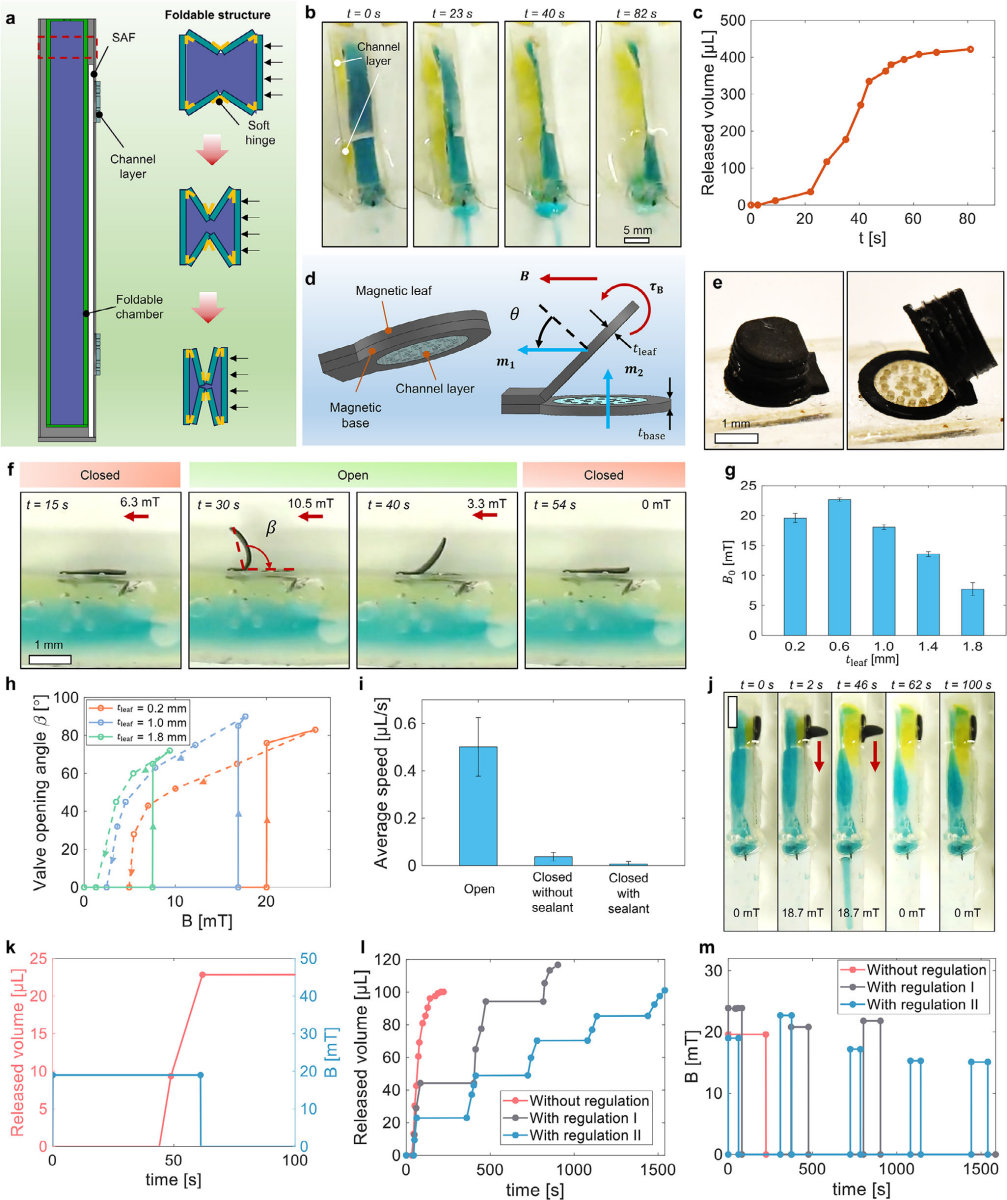
Single magnetic valve for sealing, opening, and regulating liquid release speed. (a) Illustration of the fully assembled pump, featuring a foldable drug chamber, SAF, and a channel layer on the outer shell. When pressure is applied to the foldable chamber, the loaded liquid will be ejected out. (b) Optical image of the foldable chamber and sequential images of the liquid pumping process. SAF absorbs fluid and swells to push the foldable chamber to release the loaded liquid. (c) Liquid ejection volume as a function of time during the swelling of SAF. (d) Illustration of the magnetic valve and actuation mechanism with an external magnetic field. Red, blue, and orange arrows indicate the external magnetic field, the magnetic moment of the magnetic composites, and the magnetic torque, respectively. *t*_leaf_ and *t*_base_ represent the thickness of the magnetic leaf and base, respectively. (e) Optical images depicting single‐valve control. *B* field: 6.2 mT. (f) Sequential video frames showing single magnetic valve control under an external magnetic field. (g) External magnetic fields (*B*_0_) required to open the valve with different magnetic leaf thicknesses when the magnetic field is perpendicular to the magnetic leaf magnetization. (h) Valve opening angle (β) as a function of the applied magnetic field for valves with different magnetic leaf thicknesses. (i) Average liquid ejection speed in three conditions: valve open, valve closed without sealant material, and valve closed with sealant material (multi‐purpose silicone grease, XBVV). (j) Sequential video frames showing controlled liquid release by manipulating the magnetic valve. Scale bar, 5 mm. (k) Released liquid volume and corresponding magnetic field over time. (l, m) Liquid ejection volume (l) and magnetic field magnitude (m) plotted as a function of time in three conditions: without regulation, with regulation 1 (open for 1.5 min, closed for 5 min), and with regulation 2 (open for 1 min, closed for 5 min). In j‐m, *t_leaf_* = 0.2 mm. In all cases, the magnetic base thickness *t_base_* = 0.2 mm. In g and i, error bars indicate standard deviations for *n* = 5 trials.

Precise regulation of drug dosage is essential to minimize side effects and extend drug residence time during intravesical delivery. To illustrate the fundamental mechanism of liquid release, Figure 2a shows the main structural components of the system: a channel layer with inlet ports on the outer shell, a SAF, and a foldable drug chamber. The SAF is a polymeric fiber capable of absorbing surrounding water and swelling several times its original volume, thereby generating mechanical pressure for fluid pumping. The channel layer contains hydrogel‐coated microholes (150 µm in diameter) that permit water uptake while preventing SAF extrusion. The foldable chamber is constructed using stiff Polydimethylsiloxane (PDMS) linkages connected by soft Ecoflex 00–30 hinges (Figure S1), enabling lateral deformation under the swelling‐induced pressure. As the chamber compresses, the encapsulated drug is expelled through the outlet channel. As shown in Figure 2b and Movie S1, the integrated SAF (yellow) progressively swells, exerting pressure on the foldable chamber and driving liquid ejection (blue). Quantitative analysis in Figure 2c shows that the entire release process completes within approximately 90 s.

To precisely regulate the liquid release rate and volume, a flexible magnetic valve is developed (Figure 2d). The valve is composed of a magnetic leaf, a magnetic base, and an embedded channel layer (Figures S2 and S3). Both magnetic components are magnetized perpendicular to their surfaces. In the absence of an external magnetic field, the magnetic attraction between the leaf and base keeps the valve closed. When a parallel magnetic field is applied, the induced magnetic torque deflects the leaf, opening the fluid channel. As shown in Figure 2e,f, the valve is mounted on the outer shell, where the magnetic leaf deforms to align with the external field, enabling liquid release. According to the torque balance analysis of the magnetic leaf (Experimental Sectionand Figure S4), when the magnetic torque is below a critical threshold, it cannot overcome the attractive force between the leaf and base; once the threshold is exceeded, the valve opens sharply with a large deflection angle. Upon reduction of the magnetic field, the leaf reattaches to the base, restoring the closed state through magnetic attraction. High‐speed video snapshots (Figure S5) confirm that the magnetic leaf reaches equilibrium rapidly and remains stably open or closed under the corresponding magnetic torque or leaf‐base interaction.

Furthermore, the magnetic valve can be programmed to exhibit different activation thresholds by adjusting the thickness of the magnetic leaf (Figure 2g). Increasing the leaf thickness lowers the magnetic field required for actuation, thereby extending the operational range. The thickness influences both the magnetic interaction strength and the generated torque. As shown in Figure 2h, the relationship between the valve's opening/closing angle and the applied magnetic field displays a characteristic hysteresis loop arising from magnetic coupling between the leaf and base. Thus, the valve's dynamic behavior can be customized by selecting an appropriate magnetic leaf thickness. To evaluate the triggering and sealing performance, the magnetic valve was integrated with the liquid release pump. When the applied magnetic field exceeded the threshold, the valve opened to initiate liquid release; upon field removal, magnetic attraction closed the valve to achieve sealing. The average release rate under different conditions is shown in Figure 2i, where the open‐valve configuration yields a release speed of approximately 0.5 µL s^−1^. To further improve sealing, silicone sealant containing polytetrafluoroethylene (PTFE) particles was applied along the valve edge. Without sealant, minor leakage occurred even in the closed state, whereas with sealant, water flow was completely blocked, ensuring reliable and repeatable sealing performance.

Finally, Figure 2j,k illustrate the speed‐regulated liquid release process controlled by an external magnetic field. The magnetic valve is opened by increasing the magnetic field to initiate liquid ejection and closed upon field removal to ensure reliable sealing. With stable sealing and consistent triggering performance, the overall release rate can be precisely tuned by programming the magnetic field waveform. As shown in Figure 2l,m, three actuation modes were evaluated: “Continuous opening” (no regulation), “Regulation I” (open for 1.5 min, closed for 5 min), and “Regulation II” (open for 1 min, closed for 5 min). Continuous operation released the entire liquid load within approximately 200 s. Regulation I extended the release duration to ∼900 s over three cycles, while Regulation II further prolonged it to ∼1,550 s over five cycles. These results demonstrate that by adjusting the open/close timing ratio, the drug dosage can be dynamically and precisely controlled on demand. A 7‐day in vitro drug release study was conducted to evaluate long‐term performance. Each day, liquid release was triggered by opening the magnetic valves with an external magnetic field, followed by field removal to reseal the valves. By controlling the valve opening time, a consistent release volume was achieved throughout the 7‐day study. The release process and corresponding volumes are shown in Figure S6.

### 2.3 Mechanism of Coordinating Multiple Valves for Liquid Delivery

**FIGURE 3:**
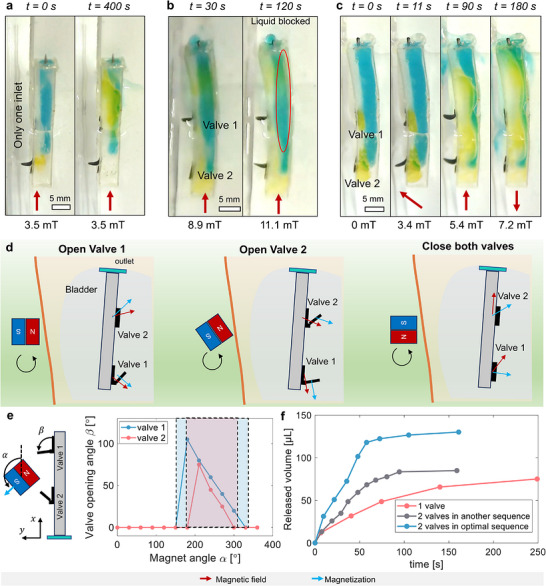
Mechanism of multiple valve coordination for controlled opening. (a) Video frames (Movie S2) of liquid ejection using only one inlet valve. (b) Video frames (Movie S2) of liquid ejection with two inlet valves opening and closing in a non‐optimal sequence. (c) Video frames (Movie S2) of liquid ejection with two inlet valves opening and closing in an optimized sequence. (d) Illustration of controlling two valves to open sequentially with a single magnet. (e) Valve opening angles β of the two valves with respect to the external magnet angle α for the optimized sequence. (f) Comparison of liquid ejection volume as a function of time for the three cases presented in (a), (b), and (c).

Coordinated control of multiple valves is essential to achieve complete and efficient drug release. While previous studies have demonstrated the use of magnetic valves for liquid manipulation in miniature soft robots [31, 32], synchronized operation of multiple valves remains a significant challenge. As shown in Figure 3a, when only a single valve is activated during liquid release, the SAF at the distal end of the patch fails to fully swell due to its distance from the inlet, leading to incomplete drug ejection, particularly in the distal region. Similarly, a non‐optimal valve activation sequence reduces efficiency (Figure 3b). For instance, if valve 2 opens first, the nearby SAF swells and compresses the adjacent chamber, impeding fluid flow when valve 1 subsequently opens. In contrast, an optimized sequence of opening valve 1 first, followed by valve 2, ensures uniform swelling of the SAF and maximizes overall drug release (Figure 3c).

Furthermore, the sequential control of dual‐valve operation was demonstrated. Figure 3d illustrates the magnetization configuration of the two valves: valve 1 is magnetized perpendicular to the valve leaf, while valve 2 is magnetized at a 45° angle relative to the normal direction. When the patch was deployed on the bladder surface, a portable magnetic actuation system equipped with permanent magnets (Figure S7) generated the required magnetic field. Initially, the permanent magnet induced a magnetic torque greater than the threshold to open valve 1 while the magnetic torque applied to valve 2 was below the threshold to maintain it closed. As the magnet rotated counterclockwise and the magnetic field rotated clockwise, the torque was progressively increased on valve 2, enabling both valves to open sequentially. Conversely, rotating the magnetic field in the opposite direction produced reverse torques that closed both valves. The optimal opening sequence was experimentally validated in Figure 3e, where the magnet's orientation corresponds to stepwise valve opening followed by simultaneous closure. Comparison of released liquid volumes under different configurations (Figure 3f) confirms that the dual‐valve system with optimized sequencing achieves the highest ejection efficiency.

### 2.4 Demonstration of Coordinating Multiple Valves for Multiplexed Liquid Delivery

**FIGURE 4:**
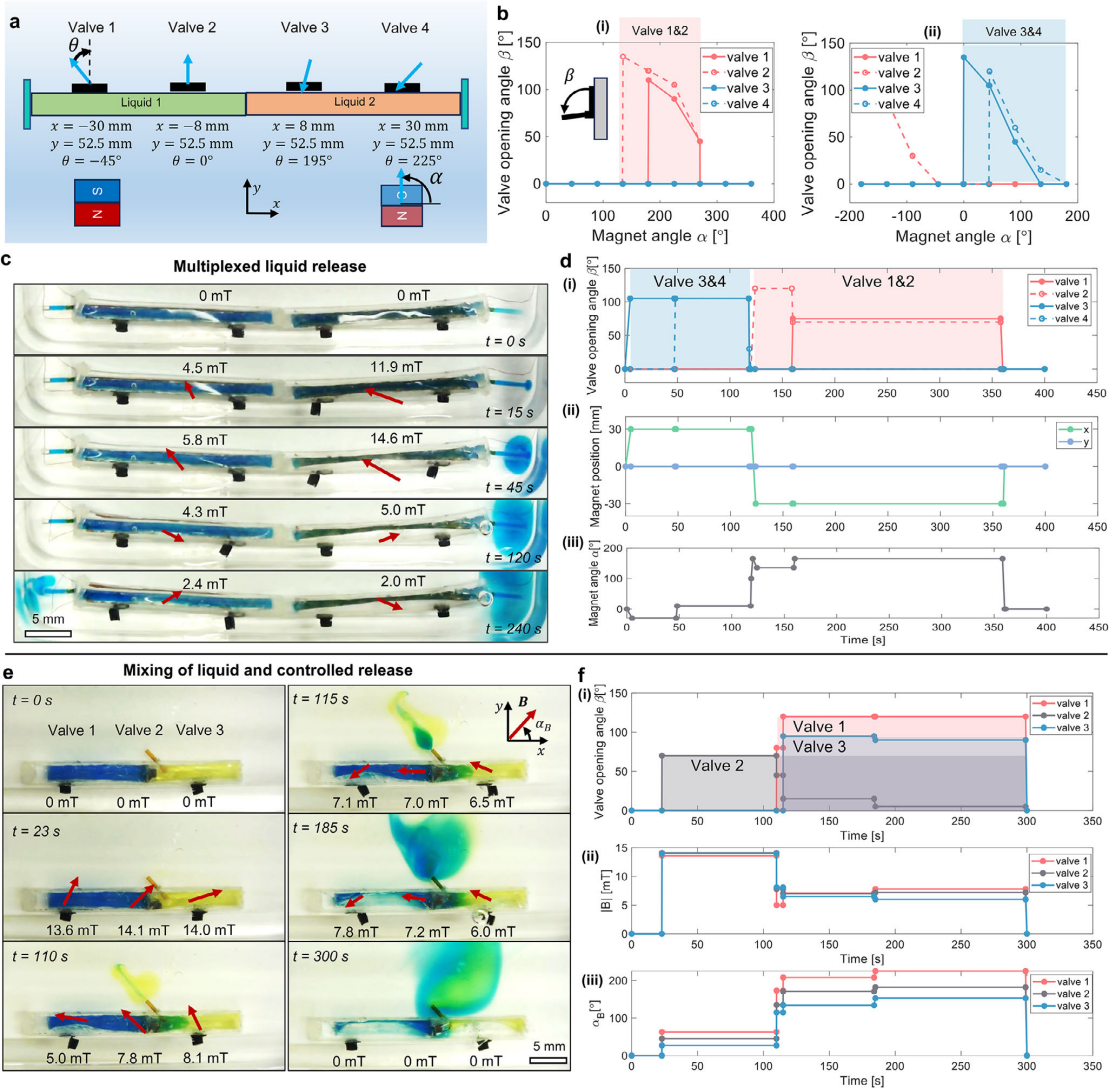
Demonstration of multiple valves coordination for controlled and multiplexed drug release. (a) Illustration of controlling four valves to open sequentially with a single magnet (25 mm by 25 mm by 25 mm, NdFeB, N45). θ is defined as the angle between the magnetization and the normal direction for the magnetic valve leaf. (b) Valve opening angles β of the four valves with respect to the external magnet angle α for the optimized sequence when the magnet is placed in front of (i) valve 1, at *x* = ‐30 mm, *y* = 0, *z* = 0, and (ii) valve 4 at *x* = 30 mm, *y* = 0, *z* = 0. (c) Video (Movie S2) frames of the sequential release for two different liquids in water controlled by external magnets. (d) Valve opening angles β (i) of the four valves, external magnet position (ii), and external magnet angle (iii) in the sequential liquid release process. (e) Video (Movie S2) frames of the mixing and release for two different liquids in water controlled by external magnets. (f) Valve opening angles β (i) of the three valves, external magnetic field magnitude (ii), and external magnetic field orientation (iii) at three valves in the liquid mixing and release process.

The opening sequence of multiple valves can be further programmed to enable multiplexed liquid release, such as sequential or simultaneous delivery of two different liquids. Figure 4a illustrates a representative configuration for dual‐liquid release, in which two SRP‐IDDs are integrated with four magnetic valves. Valves 1 and 2 regulate liquid 1, while valves 3 and 4 control liquid 2. The two valve groups are magnetized in opposite orientations to ensure that when one group opens, the other remains closed. Because the magnetic field generated by the actuation system is non‐uniform across the four valves (Figure S8), each group employs distinct magnetization directions to enable sequential activation in the optimal order. Actuation is achieved by precisely positioning and orienting the external magnets. For the release of liquid 1, the magnet is first placed near valve 1; as shown in Figure 4b, valves 1 and 2 then open sequentially while valves 3 and 4 remain closed. Conversely, positioning the magnet in front of valve 4 reverses the process, enabling sequential activation of valves 3 and 4 while valves 1 and 2 stay closed.

To further validate the dual‐liquid release functionality, the combined SRP‐IDD was immersed in water and actuated using an external magnet, as shown in Figure 4c. Valves 3 and 4 were first opened sequentially, initiating liquid release from the right patch while valves 1 and 2 remained closed to prevent unintended leakage from the left patch. Subsequently, valves 3 and 4 were closed, and valves 1 and 2 were activated to release liquid from the left patch. Figure 4d presents the corresponding valve opening and closing sequence, demonstrating the precise coordination between the two valve groups and the controlled positioning and orientation of the external magnet.

As another demonstration of multiplexed operation, a coordinated multi‐valve configuration was designed to enable in situ mixing and subsequent ejection of two liquids—potentially allowing on‐demand chemical reactions within the device. As illustrated in Figure 4e, valves 1 and 3 are positioned on the outer tube, while valve 2 is located between two foldable chambers connected by a central channel. The magnetization profiles of the three valves are engineered such that the opening plane of valve 2 is perpendicular to that of valves 1 and 3, allowing valve 2 to open first. During operation, valve 2 opens to permit one liquid to enter the chamber containing the second liquid via the central channel, achieving mixing before ejection. Valves 1 and 3 then open sequentially to expel the mixed liquid through the shared outlet positioned between the two chambers. Figure 4f depicts the corresponding valve opening angles during mixing and releasing, along with the external magnetic field strength and orientation at each valve, confirming the programmed coordination of the multi‐valve system.

### 2.5 Demonstration of Anchoring the SRP‐IDD on Biological Tissues

**FIGURE 5:**
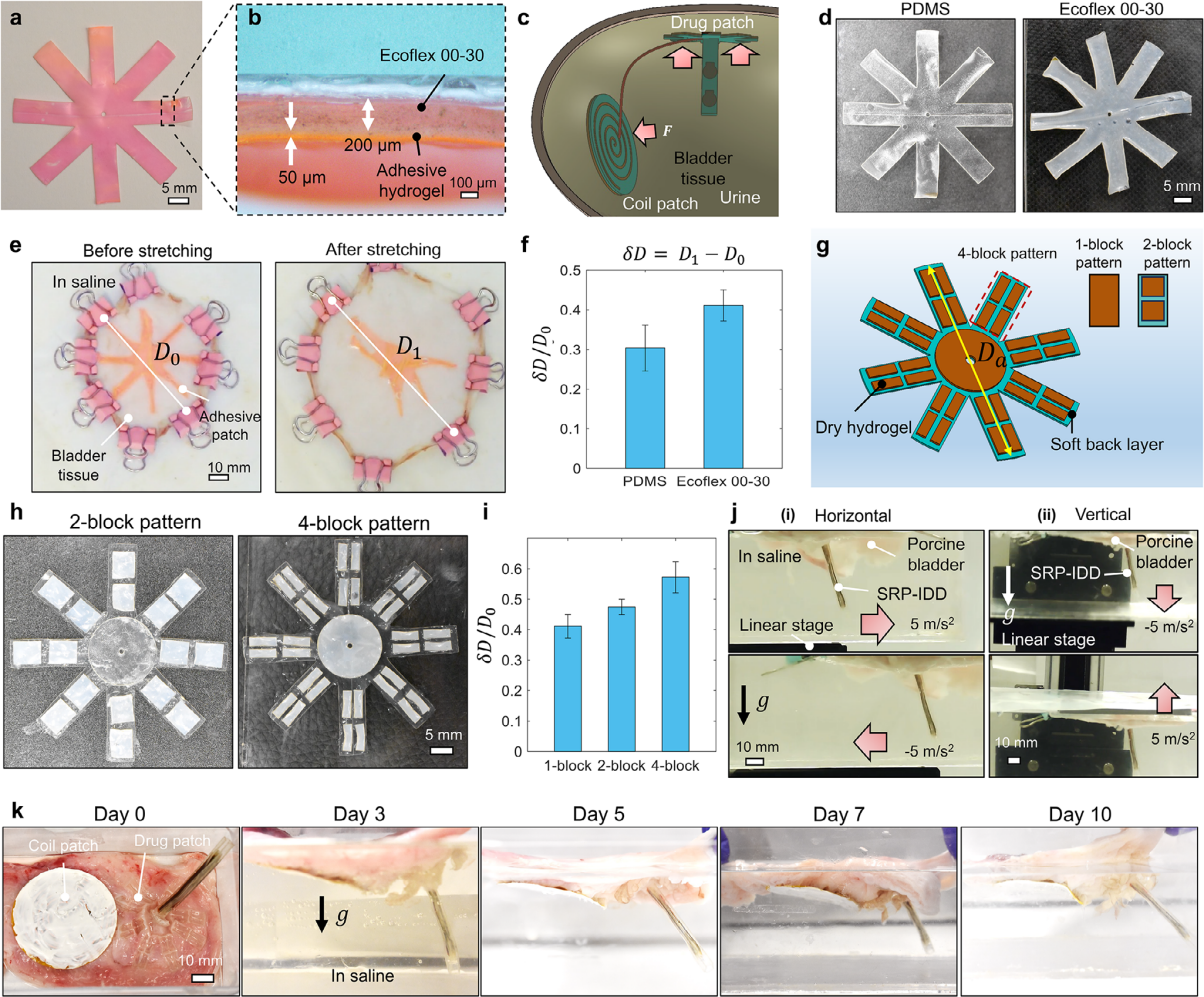
Demonstration of patterned bioadhesive enabled reliable retention of the SRP‐IDD on porcine bladder tissues. (a) Optical image of the adhesive patch. (b) Cross‐sectional image showing the thickness of the adhesive hydrogel layer and the Ecoflex 00–30 backing layer. (c) Schematic illustration of the anchoring configuration of the drug chamber and coil patch. *F* indicates the direction of the loading force. (d) Images comparing two adhesive patches with different backing layer materials made of PDMS and Ecoflex 00–30. (e) Video (Movie S3) frames of an adhesive patch on a porcine bladder tissue before and after being stretched. (f) Maximum stretching ratio ($\frac{\delta D}{D_{0}}=\frac{D_{1}-D_{0}}{D_{0}}\;$) of adhesive patches with different backing layer materials on porcine bladder tissue submerged in saline. *D*_0_ and *D*_1_ represent the initial tissue diameter and maximum tissue diameter before the adhesive patches detached, respectively. (g) Illustration of the modular adhesive patch design. (h) Optical images of the adhesive patches with different adhesive module numbers. (i) Maximum stretching ratio of adhesive patches with three distinct arm patterns on porcine bladder tissues submerged in saline (weight ratio: 0.9%). (j) Video (Movie S3) frames of testing 4‐block adhesive patch design on porcine bladder tissue (in saline) under horizontal (i) and vertical dynamic motions (ii). The acceleration of the linear stage is acquired by an IMU sensor. (k) Long‐term attachment test of the 4‐block adhesive patch design on porcine bladder tissues (in saline) over multiple days. In f and i, error bars indicate standard deviation for *n* = 5 trials.

Because the bladder is a fluid‐filled lumen, achieving reliable underwater tissue adhesion is essential for long‐term device retention. To enable stable attachment, a soft adhesive patch with a dry hydrogel interface is incorporated on the back layer of the device. The bioadhesive interface consists of a physically crosslinked poly(vinyl alcohol) (PVA) network and a covalently crosslinked poly(acrylic acid) functionalized with N‐hydroxysuccinimide ester (PAA–NHS) [35]. Upon application, the dry adhesive patch absorbs interfacial water and swells. The carboxylic acid groups in PAA–NHS enable rapid initial adhesion through physical interactions, including hydrogen bonding and electrostatic interactions [36]. Over time, covalent amide bonds form between NHS‐ester groups and primary amine groups on the bladder tissue surface, thereby providing stable and durable adhesion [35, 36]. The adhesive patch is fabricated by curing the hydrogel layer on soft substrates such as PDMS or Ecoflex 00–30 (see Experimental Section and Figure S9), then cut into a star shape for ease of catheter‐based delivery (Figure 5a). In Figure 5b, rhodamine B dye highlights the distinct thicknesses of the hydrogel layer (50 µm) and the Ecoflex 00–30 layer (200 µm). The adhesive patch is integrated with the drug‐release unit and induction coil to allow secure attachment to the bladder wall. After deployment, a controlled loading force is applied to the drug‐release and sensing patches to ensure firm anchoring (Figure 5c). The tensile test, lap‐shear test, and 180‐degree peeling test were conducted to quantify the normal adhesion strength, shear adhesion strength, and interfacial toughness of the bioadhesive patch used for the SRP‐IDD (Figure S10). For each test, the bioadhesive patch was loaded on porcine bladder tissue with the maximum force that the delivery tool typically applied, while a load cell was used to measure the adhesion.

Underwater bioadhesion enables the patch to maintain robust, long‐term retention on bladder tissues even during bladder expansion. The bladder volume can increase up to threefold during filling [37], stretching the bladder wall and posing challenges for adhesion stability. To optimize the patch design for tissue deformation, the back layer material was first investigated. As shown in Figure 5d, PDMS and Ecoflex 00–30 were selected as representative back‐layer materials. Adhesive patches were attached to porcine bladder tissues, clamped on a stretching tester to measure the maximum deformation they could withstand. During testing (Figure 5e), tissues were uniformly stretched in four directions in saline to simulate bladder expansion. The difference in tissue diameter before and after detachment (δ*D*  = *D*_1_  − *D*_0_) was measured, and the maximum stretching ratio (δ*D*/*D*_0_) was calculated. As shown in Figure 5f, adhesive patches with Ecoflex 00–30 back layers exhibited a 1.4‐fold higher maximum stretching ratio than PDMS‐based patches due to superior elasticity.

To further enhance stretchability, the hydrogel adhesive pattern was optimized. Figure 5g illustrates three modular designs for the star‐shaped patch arms: one‐block, two‐block, and four‐block patterns. As shown in Figure 5h, the two‐block pattern introduces longitudinal gaps between adhesive regions, while the four‐block pattern includes both longitudinal and transverse gaps. Systematic testing demonstrated that the four‐block design achieved the highest maximum stretching ratio (Figure 5i). The gaps allowed local deformation of the underlying soft material, reducing stress accumulation and preventing delamination during tissue stretching.

Finally, the long‐term retention of the SRP‐IDD on bladder tissue under dynamic conditions was evaluated. During human activities such as running, body acceleration induces bladder motion and urine fluctuations that could dislodge the device. To assess this, an SRP‐IDD‐loaded porcine bladder tissue was mounted upside‐down in a saline‐filled container subjected to cyclic horizontal and vertical motions using a motorized stage (Figure 5j; Figure S11). After 1 000 motion cycles at a peak acceleration of 5 m/s^2^, which exceeds the maximum acceleration (∼4 m/s^2^) experienced during normal human running [38]. The adhesive patch with the pumping unit remained firmly attached. The retention time of the SRP‐IDD in saline was further assessed, as shown in Figure 5k. The device maintained strong adhesion to bladder tissue for up to 10 days underwater.

### 2.6 Demonstration of Controlled Drug Release in Ex Vivo Organs by a Portable Magnetic Actuation Unit

**FIGURE 6:**
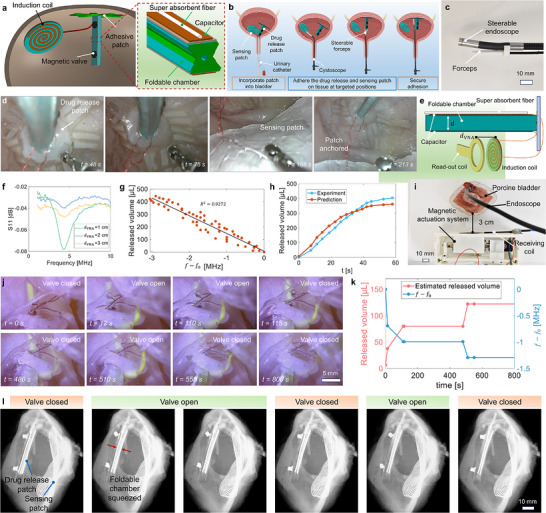
Delivery, anchoring, release control, and drug release volume monitoring of the SRP‐IDD in an ex vivo porcine bladder. (a) Illustration of the SRP‐IDD on a bladder tissue with a magnified view highlighting the foldable chamber for release and volume sensing. (b) Illustration of the process of delivering and anchoring the SRP‐IDD in a bladder using a urinary catheter, a cystoscope, and steerable forceps. (c) Optical image of the customized delivery tool combining a steerable endoscope and forceps. (d) Video (Movie S4) frames showing the patch delivery and anchoring process in an ex vivo porcine bladder. (e) Illustration of the LC circuit‐based sensing mechanism for detecting drug release volume. (f) S_11_ parameter vs. frequency at varying detection distances when the sensor is placed in saline and attached to porcine bladder tissues. (g) Resonance frequency variation vs. released liquid volume during the calibration process. (h) Comparison of measured and predicted liquid release volumes over time. (i) Optical image of the experimental setup for controlled liquid release in a porcine bladder using a portable magnetic actuation system and a VNA‐based drug release volume sensing system. (j) Video (Movie S5) frames showing controlled drug release and sensing of the SRP‐IDD in a porcine bladder. (k) Normalized frequency and predicted released liquid volume as a function of time in (j). (l) X‐ray images capturing the controlled liquid release process of the SRP‐IDD in a porcine bladder.

First, to demonstrate the liquid release functionality of the SRP‐IDD, the delivery procedure and controlled drug release were validated in ex vivo porcine bladders. Figure 6a presents the integrated architecture, which includes magnetic valves for regulating liquid release, a swelling‐based pump for drug ejection, an LC circuit for monitoring release volume, and an adhesive patch for stable tissue anchoring. The device can be delivered through a urinary catheter, enabling minimally invasive deployment into the bladder. The stepwise delivery process is illustrated in Figure 6b. After urine expulsion, both the drug‐release patch and the sensing patch are introduced into the bladder via a urinary catheter. A cystoscope equipped with forceps guides the drug‐release patch to the target site. Under cystoscopic imaging, the patch is grasped and attached to the upper bladder wall to prevent stimulation of the lower bladder that could induce unintended urination. The sensing patch is positioned on the anterior bladder wall to minimize the distance to the skin surface and improve signal transmission. Once positioned, controlled loading forces are applied via the forceps to ensure secure adhesion.

Then, to replicate the clinical delivery procedure in a controlled laboratory setting, a customized benchtop tool was developed (Figure 6c), consisting of a steerable endoscope and forceps mounted using 3D‐printed fixtures. Using this setup, the full procedure was demonstrated in ex vivo porcine bladders (Figure 6d). Both patches were introduced through the catheter, and the drug‐release patch was grasped and guided to the bladder's upper wall. Each adhesive arm was flattened and pressed onto the tissue surface to achieve stable attachment. Finally, the sensing patch was placed at its designated location and anchored for consistent positioning.

In addition, to enable real‐time monitoring of drug release, an LC circuit was integrated with the foldable chamber. As shown in Figure 6e and Figure S12, two capacitor plates were affixed to the flat sides of the chamber. During drug release, chamber compression reduced the plate separation, thereby increasing capacitance (Figure S13). As shown in Figure S14, capacitance increased proportionally with liquid release, demonstrating good repeatability. The capacitor was connected to an induction coil, forming an LC circuit whose resonant frequency decreased with rising capacitance. The reflection coefficient (S_11_ parameter), which defines the ratio of incident and reflected waves from a port, was wirelessly measured using a vector network analyzer (VNA), with the peak frequency corresponding to the LC circuit resonance. The capacitive sensor, induction coil, and connecting wires were encapsulated with Ecoflex 00–30 to ensure sealing and secure connections. Mechanical fatigue testing using a motorized linear stage showed stable connections and unchanged signal quality after 1000 stretching cycles. In a tissue‐mimicking test, the SRP‐IDD mounted on porcine bladder tissue also withstood 1000 cycles of stretching, remaining intact with both patches firmly attached. Consistent S_11_ measurements before and after testing further confirmed electrical stability (Figure S15).

Furthermore, Figure 6f shows that the wireless sensing system reliably detected signals at distances up to 3 cm when the induction coil was deployed on porcine bladder tissue submerged in saline. Signal quality was further improved using a larger induction coil with higher inductance (Figure S16). A predictive model correlating resonant frequency shifts with released drug volume was then established (Note S2 and Figure S17). To eliminate environmental effects from surrounding tissue and fluids, the resonant frequency was normalized by its initial value, as relative frequency changes consistently tracked capacitance variation. A linear fit between normalized resonant frequency and released volume yielded a strong correlation (Figure 6g), allowing accurate prediction of liquid release from VNA measurements. To validate the sensing function, Figure 6h compares the predicted and experimentally measured drug volumes over time, showing close agreement between the two. As shown in Figures S18 and S19, the VNA wirelessly monitored the LC circuit's resonant frequency while the released liquid was collected through a thin plastic tube. The gradual decrease in normalized resonant frequency provided a reliable indicator of drug release volume, confirming the effectiveness of wireless sensing for quantitative monitoring.

After deployment of the SRP‐IDD, controlled liquid release was demonstrated in an ex vivo porcine bladder filled with saline. Figure 6i shows the experimental setup, in which the bladder was sutured onto a 3D‐printed phantom for stable handling. An endoscope integrated through the urethra, equipped with an ultraviolet (UV) light emitting diode (LED), provided clear visualization of the released liquid, while fluorescent dye loaded in the patch enabled real‐time tracking. A portable magnetic actuation system, coupled with a receiving coil connected to a VNA, was used for both magnetic actuation and wireless sensing, which is an approach that could be readily adapted for patient use at home. As shown in Figure 6j, both the drug‐release and sensing patches were deployed and submerged in saline. Initially, the magnetic valves remained closed, and the S_11_ signal exhibited a stable resonance peak at 7.89 MHz. When the magnetic actuation system was activated, the rotating magnets generated torque that opened the valves, triggering liquid release. The fluorescent dye was clearly visualized under endoscopy, while the S_11_ resonance peak shifted leftward from 7.89 MHz to 6.90 MHz. When the magnets were rotated back, the valves closed, halting liquid release and allowing gradual dye diffusion, with the S_11_ peak stabilizing. Reapplying the magnetic field reopened the valves, leading to renewed liquid release and a further frequency shift to 6.60 MHz. Once the valves were closed again, no additional frequency changes were detected, confirming complete sealing of the device. Based on the calibration model established previously, the released liquid volume was quantitatively predicted from the normalized resonant frequency, as shown in Figure 6k.

Lastly, following SRP‐IDD deployment, medical imaging modalities such as X‐ray imaging can also be used to validate the device status and monitor liquid release. To evaluate this capability, the SRP‐IDD was attached to a porcine bladder (Figure 6l) after loading the drug chamber with an X‐ray contrast agent. The X‐ray images clearly revealed the magnetic valves, capacitor‐integrated foldable chamber, and sensing patch. Upon magnetic actuation, the valve was observed in its open state, and the distance between the two capacitor plates decreased, indicating drug ejection. Sequential X‐ray imaging further confirmed that the contrast agent was gradually released while the valves remained open, demonstrating that the entire process can be continuously tracked to validate device function and release behavior in real time.

## 3 Discussion

In summary, we have developed an SRP‐IDD that enables remotely controlled, multiplexed, and sustained drug delivery. The system integrates multiple magnetic valves to regulate a soft robotic pump that harnesses osmotic pressure for precise liquid dosing. To ensure stable attachment, the device incorporates a bioadhesive‐based soft patch that can be catheter‐delivered for minimally invasive deployment. An integrated resonance‐frequency LC circuit enables real‐time monitoring of liquid release, ensuring accurate dosage control, while a portable magnetic actuation unit allows fully wireless operation through selective regulation of multiple valves. The device's functionality and reliability were systematically validated in phantom models and ex vivo porcine bladders, confirming robust and repeatable performance.

This noninvasive SRP‐IDD offers a promising approach for on‐demand and long‐term intravesical drug delivery, particularly for treating bladder cancer and IC. The PDMS encapsulation ensures biocompatibility during prolonged bladder contact, and the compact, flexible structure minimizes discomfort while facilitating minimally invasive application. By conforming to the bladder surface via integrated bioadhesives, the patch maintains a stable mechanical interface that enables controlled, sustained, and localized drug release. For clinical application, the SRP‐IDD can be squeezed into a urinary catheter for delivery to the bladder to form stable anchoring on biological tissues using the adhesive patch and then filled with a drug. Patients can use a portable magnetic actuation system to control the magnetic valve and acquire drug release volume wirelessly using the wireless sensing unit. Collectively, this platform represents a significant step toward long‐term, programmable treatment strategies capable of reducing morbidity and improving quality of life for patients with urological diseases.

Despite the promising results, several limitations remain to be addressed in future work. First, refilling the device is currently challenging due to the use of a superabsorbent fiber‐based pump. This limitation could be overcome by adopting alternative pumping mechanisms, such as magnetic screw–piston pumps [39], which allow pumping and refilling controlled by magnetic fields, and elastic‐based pumps with refilling tubes shown in Figure S20, which can still be controlled by the magnetic valve mechanism for multiplexed liquid release in bladder. Second, the retention time of the device is constrained by the bioadhesive formulation, which currently supports stable attachment for approximately one to two weeks. The use of an advanced electroadhesive hydrogel interface [40]. or underwater mechanical adhesives [41] may further extend retention duration for long‐term implantation. In addition, incorporating mechanical retention structures [42] could further improve device stability and residence time. At present, the device primarily monitors drug release volume. Future integration of multimodal flexible sensors could allow simultaneous measurement of physiological parameters such as pH [43], pressure [37], and tissue strain or stiffness [37, 44], enabling real‐time, closed‐loop therapeutic control [24, 45, 46]. In addition, incorporating bistable structures into the pump and valve design [47]. could facilitate more complex, programmable, and multiplexed liquid release, expanding the device's functionality for future biomedical applications.

To further validate long‐term drug release performance and device retention, in vivo studies in large animal models are planned. For in vivo validation, several challenges remain to be addressed. First, host immune responses may induce acute inflammation or chronic cystitis, necessitating mitigation strategies such as anti‐inflammatory surface coatings or localized drug delivery. Second, the complex urinary environment may promote biofouling, which can impair adhesion performance and drug release, thereby motivating antifouling and antimicrobial modifications. In addition, variations in urine composition, including fluctuations in pH, ionic strength, and metabolite concentrations, may affect device stability and must be systematically evaluated under physiologically relevant conditions. Third, although the functionality of the SRP‐IDD has been demonstrated for 7 days, long‐term operation over weeks to months will require further optimization through more robust adhesive interfaces and refillable reservoir designs.

In the future, the device diameter (∼3 mm) will be further miniaturized to ensure compatibility with the working channel of a clinical cystoscope. The magnetic field required to operate the valves is below 20 mT, which is generally considered safe without harmful biological effects [48]. However, it may interfere with implantable devices such as pacemakers or implantable cardioverter‐defibrillators. The magnetic materials used are also not compatible with magnetic resonance imaging (MRI) due to the strong magnetic fields. Future efforts will also focus on assessing and optimizing the device's compatibility within MRI environments to accommodate patients requiring MRI‐based diagnostics.

Overall, the proposed system presents a promising platform for controllable, localized drug delivery in confined anatomical spaces. The integration of wireless sensing enables closed‐loop therapeutic regulation, while the use of magnetically actuated valves allows on‐demand, programmable, and sequential drug release. This minimally invasive strategy offers significant potential for long‐term management of chronic diseases and personalized treatment in internal organs.

## 4 Experimental Section

### 4.1 Preparation of Magnetic Soft Valves

The magnetic soft valve was composed of a magnetic leaf, a magnetic base made by magnetic composites, and a channel layer made by PDMS. Magnetic composites were fabricated by mixing Ecoflex 00–30 silicone rubber with NdFeB microparticles (average diameter, 5 µm; MQFP‐15‐7, Neo Magnequench) at a 1:2 weight ratio. The mixed material was poured and scraped on a glass substrate with a 200 µm spacer and cured on the hot plate at 70°C for 20 min. The magnetic leaf and base were cut by the LPKF U4 protolaser machine (LPKF Laser & Electronics North America) and then magnetized in perpendicular direction with 2.61 and 0.74 T magnetic fields by an impulse magnetizer (IM‐10‐30, ASC Scientific), respectively. A PDMS patch was prepared on a glass substrate with 200 µm spacer. The channel layer with 150 µm holes was then cut with the PDMS patch by the LPKF U4 protolaser machine. The channel layer was embedded in the magnetic base layer by bonding the edge with PDMS. The magnetic leaf was attached to the base only on the square part by PDMS in a configuration where the leaf and base attracted each other. The assembled magnetic soft valve was then glued on the hole of the outer shell.

To allow liquid to pass through, a hydrogel coating was performed for the channel layer. Benzoyl peroxide (Sigma–Aldrich Inc.) was first dissolved in acetone at 10 wt.% (Sigma–Aldrich Inc.). Next, a PEGDA solution (20 wt.% in water, Sigma–Aldrich Inc.) was prepared with ammonium persulfate (1 wt.%, Sigma–Aldrich Inc.). An external magnetic field was applied to open the valve, and the benzoyl peroxide solution was first pipetted onto the channel layer. After 2 min, the PEGDA solution was added and heated with a hot air gun at 70°C until the crosslinking of the hydrogel forms on the polymer surface. Finally, the solid hydrogel was removed from the channels.

### 4.2 Control of the Opening and Closing of Multiple Magnetic Valves

To enable the simultaneous control of multiple valve operations, it is essential to characterize the opening and closing behavior of the magnetic valves. The magnetic leaf can be modeled as a rigid body connected to a fixed magnetic base through a flexible joint. For simplicity, the valve actuation process is treated as quasi‐static. As illustrated in Figure S4, based on the torques acting on the magnetic leaf, a torque balance equation along the *x*‐axis can be derived as follows:

(1) $$\tau_{B}^{\mathrm{ext}}+\tau_{B,1}^{\mathrm{inter}}+\tau_{\mathrm{Bg},1}^{\mathrm{inter}}+\tau_{e}+\tau_{n}=0$$

where $\tau_{B}^{\mathrm{ext}}$ represents the magnetic torque induced by the external magnetic field, $\tau_{B,1}^{\mathrm{inter}}$ represents the magnetic torque induced by the magnetic field generated by the magnetic base, $\tau_{\mathrm{Bg},1}^{\mathrm{inter}}$ represents the torque induced by the magnetic gradient force between the magnetic leaf and base, and *τ*_**e**_ represents the torque due to elastic deformation of the leaf. In addition, *τ*_**n**_ represents the torque induced by the reaction force applied by the magnetic base. The external magnetic torque can be calculated as

(2) $$\tau_{\mathrm{ext}}^{B}=m_{2}\times B_{ext}^{yz}$$

where ***m***_2_ is the magnetic moment of the magnetic leaf and $B_{\mathrm{ext}}^{xyz}$ is the external magnetic field controlled by the external magnet position and orientation, which is considered identical across the entire magnetic valve. To calculate the magnetic torque by the magnetic base, the magnetic leaf is discretized. For the *j*‐th element (*j* = 1, …, *N*_2_), the magnetic torque induced by the magnetic field generated by the magnetic base can be calculated as

(3) $$\tau_{B,1j}^{i\mathrm{nter}}=m_{2j}\times B_{1}\left(r_{2j}\right)$$

where ***m***_2**j**_ is the magnetic moment of the *j*‐th element of the magnetic leaf and ***B***_1_(***r***_2*j*_) is the magnetic field generated at the *j*‐th element location by the magnetic base. To calculate the magnetic field generated by the magnetic base, the magnetic base is first discretized, and then a magnetic dipole model is used to calculate the magnetic field at the *j*‐th element of the magnetic leaf, given by

(4) $$B_{1}\left(r_{2j}\right)\;=\int_{\Omega_{1}}\frac{\mu_{0}}{4\pi }\left[\frac{3\left(r_{2j}-r_{1i}\right)\left(m_{1i}\cdot \left(r_{2j}-r_{1i}\right)\right)}{{\left|r_{2j}-r_{1i}\right|}^{5}}-\frac{m_{1i}}{{\left|r_{2j}-r_{1i}\right|}^{3}}\right]\;$$

where *m*_1**i**_ is the magnetic moment of the *i*‐th (*i* = 1, …, *N*_1_) element of the magnetic base, *r*_1**i**_ and *r*_2**j**_ are the position vectors of *i*‐th element of the magnetic base and *j*‐th element of the magnetic leaf, respectively. μ_0_ is the magnetic permeability of air, and **Ω**_1_ represents the domain of magnetic base. The total magnetic torque is given by

(5) $$\begin{array}{cc} \tau_{\mathrm{inter}}^{B,1} & =\int_{\Omega_{2}}\tau_{\mathrm{inter}}^{B,1j}\;=\int_{\Omega_{2}}m_{2j}\\ & \times \int_{\Omega_{1}}\frac{\mu_{0}}{4\pi }\left(\frac{3\left(r_{2j}-r_{1i}\right)\left(m_{1i}\cdot \left(r_{2j}-r_{1i}\right)\right)}{{\left|r_{2j}-r_{1i}\right|}^{5}}-\frac{m_{1i}}{{\left|r_{2j}-r_{1i}\right|}^{3}}\right)\; \end{array}$$

where **Ω**_2_ represents the domain of the magnetic leaf. For the *j*‐th element of the magnetic leaf, the torque induced by the magnetic attraction between the magnetic leaf and base can be calculated as

(6) $$\tau_{\mathrm{Bg},\;1j}^{\mathrm{inter}}=r_{2j}\;\times \;F_{1,2j}=r_{2j}\;\times \int_{\Omega_{1}}F_{1i,2j}$$

where *F*_1**i**,2**j**_ is the force between the *i*‐th element of the magnetic base and the *j*‐th element of the magnetic leaf, which can be simplified as the force between two magnetic dipoles. Therefore, we have

(7) $$\begin{array}{cc} F_{1i,2j} & =\frac{3\mu_{0}}{4\pi {\left|r_{2j,1i}\right|}^{5}}[\left(m_{1i}\cdot r_{2j,1i}\right)m_{2j}+\left(m_{2j}\cdot r_{2j,1i}\right)m_{1i}\\ & \;+\left(m_{1i}\cdot m_{2j}\right)r_{2j,1i}-\frac{5\left(m_{1i}\cdot r_{2j,1i}\right)\left(m_{2j}\cdot r_{2j,1i}\right)}{{\left|r_{2j,1i}\right|}^{2}}r_{2j,1i}] \end{array}$$

where *r*_2**j**,1**i**_ = *r*_2**j**_  − *r*_1**i**_ is the position vector between the *i*‐th element of the magnetic base and the *j*‐th element of the magnetic leaf. The total torque induced by the magnetic attraction can be calculated as

(8) $$\tau_{\mathrm{inter}}^{\mathrm{Bg},\;1}=\int_{\Omega_{2}}\left(r_{2j}\times \int_{\Omega_{1}}F_{1i,2j}\right)\;$$

As the distance between the magnetic leaf and base increases, *τ*_**B**1,**j**_ and *τ*_**m**,**j**_ will decrease rapidly. The torque due to elastic deformation of the leaf is simplified as a torsional spring, which can be calculated as

(9) $$\tau_{e}=k\left(\beta -\beta_{0}\right),$$

where ***k*** is a constant vector along *x* axis, and *β* is the opening angle of the magnetic leaf. *β*_0_ is zero when there is no prestress.

In the opening process of the magnetic valve, the magnetic interaction induced torques $\tau_{B,1}^{\mathrm{inter}}$ and $\tau_{\mathrm{Bg},1}^{\mathrm{inter}}$ remain constant when the valve is closed. The elastic stress‐induced torque *τ*_**e**_ is equal to zero as the magnetic leaf has no elastic deformation initially. When the external magnetic torque gradually increases, the reaction torque τ_**n**_ gradually decreases. When the magnetic valve is at the critical point to open, the reaction torque ***τ***_**n**_ becomes zero. The critical torque required to open the valve is given by,

(10) $$\tau_{B}^{\mathrm{ext}}\;\left(B_{\mathrm{yz}}^{\mathrm{ext}}\right)=-\tau_{B,1}^{\mathrm{inter}}-\tau_{\mathrm{Bg},1}^{\mathrm{inter}},$$

determined by the geometry of the valve and magnetization of the magnetic leaf and base.

By designing the magnetization of the magnetic leaf and tuning the applied magnetic torque by adjusting the position and orientation of the actuation magnet, sequential opening of the valves can be achieved. After the valve is open, the torques $\tau_{B,1}^{\mathrm{inter}}$ and $\tau_{\mathrm{Bg},1}^{\mathrm{inter}}$ decrease rapidly and can be neglected when the distance between the magnetic leaf and base increases. The torque balance equation can be simplified as $\tau_{\mathrm{ext}}^{B}\left(B_{\mathrm{ext}}^{yz}\right)=k\beta$, while the magnetic leaf maintains a constant opening angle with a static external magnetic field. In the closing process of the magnetic valve, as the applied external magnetic torque reduces, the opening angle will also decrease due to the torque balance. The torques τ_**B**1,**j**_ and τ_**m**,**j**_ gradually increase in this procedure. When $|\tau_{\mathrm{ext}}^{B}\left(B_{\mathrm{ext}}^{yz}\right){|}_{x}<|\tau_{\mathrm{inter}}^{B,1}+\tau_{\mathrm{inter}}^{\mathrm{Bg},1}+\tau_{e}{|}_{x}$, the valve closes. Other factors that warrant further consideration include interfacial adhesion, which may introduce discrepancies in the model that can be compensated by an offset term. In addition, PTFE paste particles may contribute not only to adhesion and capillary effects but also to filling gaps at the interfacial surfaces.

### 4.3 Preparation of the Foldable Drug Chamber and Outer Shell

The foldable drug chamber and outer shell were fabricated with PDMS (Dow Silicones Corporation) and Ecoflex 00–30 silicone rubber (Smooth‐On Inc.). PDMS patches were prepared with a 10:1 weight ratio of monomers to cross‐linker on glass substrates with 50 and 400 µm spacers, respectively. An Ecoflex 00–30 patch was prepared with 50 µm spacer. To assemble the foldable drug chamber, 2 mm wide and 1 mm wide rectangular sheets were cut by a LPKF ProtoLaser U4 with 50 µm thick PDMS patch as linkages and 1 mm wide Ecoflex sheets of the same length as hinges. Two 1 mm and one 2 mm wide PDMS sheets were placed side by side and two Ecoflex sheets were glued on top of the gap with PDMS. Two identical units were then connected with two Ecoflex sheets on the other side to form a chamber. A square top cap and bottom cap with outlets were cut with the Ecoflex patch and glued to the chamber. The outer shell was assembled with 4 rectangular 400 µm PDMS sheets cut by LPKF, one of which was cut two holes for the channel layers.

### 4.4 Preparation of the LC Circuit for Released Liquid Volume Sensing

The LC circuit was composed of a capacitor attached to the foldable drug chamber and an induction coil. Etching masks were patterned on a piece of pyralux (DuPont) covered by polyimide (PI) tape with two rectangles for capacitor plates and a spiral trace designed in SolidWorks for the induction coil by LPKF U4 protolaser machine. The patterned pyralux was then submerged in ferric chloride (MG Chemicals) for etching after the mask was removed. Afterwards, the two plates of the capacitor were connected to the two ends of the induction coil by soldering two copper wires. Ecoflex 00–30 was applied to the capacitor and inductor for insulation. Two plates of the capacitor were attached to the two flat sides of the foldable chamber with PDMS.

### 4.5 Experimental Setup for Sensing

A VNA (LibreVNA) was used to acquire signals from the LC circuit. The drug release patch and induction coil were first attached to the porcine tissue and later submerged in water. A read‐out coil was connected to one of the ports on VNA, which was placed around the tissue and aligned with the induction coil. S11 parameter was recorded across the entire drug release process, and its peak frequency was analyzed to predict the released volume.

### 4.6 Fabrication of the Adhesive Patch

The stock solution of the adhesive was prepared by adding 35 w/w% acrylic acid (AAc, Thermo Fisher Scientific Inc.), 7 w/w% polyvinyl alcohol (98‐99% hydrolyzed, high molecular weight, Thermo Fisher Scientific Inc.), 0.2 w/w% α‐ketoglutaric acid (Chem‐Impex Int'l Inc.), and 0.05 w/w% N, N’‐bis(acryloyl)cystamine (Thermo Fisher Scientific Inc.) to nitrogen purged deionized water. The mixture was stirred at 90°C until the solid fully dissolved. 30 mg of N‐acryloxysuccinimide (AAc‐NHS, Thermo Fisher Scientific Inc.) was added to every 1 mL stock solution for polymerization. An Ecoflex 00–30 patch of 200 µm thickness was prepared as a base layer and treated with 10w/v% benzophenone (Sigma–Aldrich) in ethanol for 10 min. The hydrogel precursor was applied to the treated Ecoflex 00–30 patch and then capped by a glass slide with 50 µm spacer, which was covered by polyethylene terephthalate (PET) tape for easier demolding. The adhesive was then placed in a UV chamber (365 nm, 15 W) for 30 min. The cured adhesive hydrogel with Ecoflex 00–30 base was dried under air flow for 3 h and cut into the desired shape with the LPKF protolaser machine. Afterwards, the adhesive hydrogel modules were attached on a 200 µm thick Ecoflex 00–30 base using uncured Ecoflex 00–30 at room temperature. Lastly, the adhesive patches were integrated with the drug release chamber and the induction coil by Ecoflex 00–30.

### 4.7 Portable Magnetic Actuation System

The portable magnetic actuation system was a two‐degree‐of‐freedom (DOF) system and had a relatively small component size compared to the clinic‐used magnetic actuation system. It consisted of one magnetic actuator comprising two magnets (N45 grade 25 mm cube NdFeB magnets), a bipolar stepper motor (17HS24‐2104S), a motor driver (TC1508A Mini Module), a 9 V battery (Quzmo 9 V 1300 mAh rechargeable Li‐ion Battery) as a power supply, and one Arduino controller (Arduino Nano BLE 33). The stepper motors were driven by motor drivers, which were in turn controlled by the Arduino controller. Translational motion was manually guided by slide rails and guide wheels, while rotational motion was controlled by the Arduino and driven by the stepper motor. The Arduino received control messages via Bluetooth from a PC, parsed the commands, and executed angle or speed control for the stepper motor and magnetic actuator. This system enabled control of the motion of a permanent magnet, allowing adjustment of the magnitude and direction of the external magnetic field.

### 4.8 Test the Liquid Release on Porcine Bladder Tissues

To prepare the liquid‐loaded SRP‐IDD, SAF was twisted to a long bundle and then integrated into the chamber between the outer tube and the foldable drug chamber. Fluorescein green (365 nm wavelength, Aldon corporation) was loaded into the drug chamber from the outlet on the bottom cap with a syringe. For testing the liquid release, a porcine bladder was sutured on a 3D‐printed soft bladder phantom. The liquid‐loaded SRP‐IDD with an induction coil was first delivered and anchored on the bladder tissue. An endoscope (Teslong) bundled with a UV LED (12 V, EDGELEC) was deployed in the bladder for visualization. A customized portable magnetic actuation system was used to provide an external magnetic field to actuate the magnetic valves. A read‐out coil connected with a VNA was integrated with the portable magnetic actuation system to acquire signal from the induction coil deployed in the porcine bladder.

### 4.9 Procedure of the Dynamic Motion Test

To test the retention under dynamic motion, an SRP‐IDD was first loaded on a piece of porcine bladder tissue. The bladder tissue was then fixed on a container upside‐down when submerged in saline. The container was attached to a motion stage to provide both horizontal and vertical motions. An IMU sensor (MPU‐6050) was also fixed on the motion stage to measure the acceleration of the motion. 1000 cycles of horizontal and vertical motion were induced with a maximum acceleration of 5 m/s^2^, and the medical retention was validated after the testing.

### 4.10 Procedure for Testing the Retention Time

To test the retention time of the adhesive patch, a drug release patch, and a sensing patch were loaded on a piece of porcine bladder tissue. The tissue was submerged in saline, which was replaced every day with the patch anchored upside down. The patch anchoring status was monitored until it was detached.

### 4.11 Procedure of Delivering the Device Using an Endoscope With Forceps

To deliver the SRP‐IDD on bladder tissue, a pair of forceps (Cook Medical Inc.) was used for deployment, which was fixed with an endoscope for visualization. The drug release patch with the sensing patch was first integrated into the bladder through the urethra. Then, the drug release patch and the sensing patch were grabbed by the forceps and loaded on the top and front part of the bladder, respectively. Lastly, both the drug release patch and sensing patch were pressed by the forceps to secure the anchoring on the bladder tissue.

## Author Contributions

Conceptualization was performed by X.D. Methodology was carried out by X.D. and B.X. Investigation was conducted by B.X., Y.Z., and Y.W. Visualization was completed by B.X. and X.D. Supervision was provided by X.D. The original draft was written by X.D. and B.X, and the manuscript was reviewed and edited by X.D., B.X., J.P., and J.T.

## Conflicts of Interest

Vanderbilt University has filed a provisional patent application related to this work. The authors declare that they have no other competing interests.

## Supporting information




**Supporting File 1**: adhm71197‐sup‐0001‐SuppMat.pdf.

**Supporting File 2**: adhm71197‐sup‐0002‐MovieS1.mp4.

**Supporting File 3**: adhm71197‐sup‐0003‐MovieS2.mp4.

**Supporting File 4**: adhm71197‐sup‐0004‐MovieS3.mp4.

**Supporting File 5**: adhm71197‐sup‐0005‐MovieS4.mp4.

**Supporting File 6**: adhm71197‐sup‐0006‐MovieS5.mp4.

## Data Availability

The data that supports the findings of this study are available in the supplementary material of this article.
