# Supplementary material for: Fully Wireless and Flexible Valves for Multiplexed and Prolonged Intravesical Liquid Release
Source: Adv Healthc Mater. 2026 May 7;15(23):e71197. doi: 10.1002/adhm.71197 (PMC13280199; doi:10.1002/adhm.71197)
Supplement: Supplementary file 1 — Supporting File 1: adhm71197‐sup‐0001‐SuppMat.pdf. [file ADHM-15-0-s006.pdf]

Supporting Information for

Fully Wireless and Flexible Valves for Multiplexed and Prolonged Intravesical Liquid Release

Boyang Xiao[1], Yi Zhu[1], Yusheng Wang[1], Janene M. Pierce[4,5], Jeffrey J. Tosoian[4,5], Xiaoguang Dong[1,2,3,*]

[1] Department of Mechanical Engineering, Vanderbilt University, TN 37212, US
[2] Department of Biomedical Engineering, Vanderbilt University, TN 37212, US
[3] Vanderbilt Institute for Surgery and Engineering, Vanderbilt University, TN 37212, US
[4] Department of Urology, Vanderbilt University Medical Center, Nashville, TN, 37232 US
[5] Vanderbilt-Ingram Cancer Center, Nashville, TN, 37232 US

* Corresponding to xiaoguang.dong@vanderbilt.edu

The PDF file includes:

Fig. S1 to S20
Supplementary Note 1
Notes for movies S1 to S5

Other Supplementary Material for this manuscript includes the following:

movies S1 to S5

Supporting Information Text

Supplementary Note 1. Resonance Frequency of the Sensing Circuit.

The liquid release volume sensing mechanism is based on an LC circuit composed of a parallel-plate capacitor integrated with the foldable chamber (**Fig. S13**) and an induction coil for data transmission. Two capacitor plates are attached on the top and bottom sides of the foldable chamber respectively. The capacitance can be calculated as

$$C = \frac{\varepsilon_r \varepsilon_0 A}{d} = \frac{\varepsilon_r \varepsilon_0 l_f w_f}{d}, \quad (1)$$

where ε_r is relative permittivity decided by the substrate, foldable chamber wall and loaded liquid; ε_0 is permittivity of free space; A is the overlapping area of the capacitor plates; l_f is the length of the foldable chamber; w_f is the width of the foldable chamber; d is the separation distance between the capacitor plates. When the SAF-based pump starts swelling, the foldable chamber deforms, resulting in loaded liquid release and capacitance change. From the geometry constraint, the released volume can be calculated as

$$V_r = V_0 - V = w_f d_0 l_f - \left(w_f d - \frac{1}{2} d\sqrt{d_0^2 - d^2}\right) l_f, \quad (2)$$

where V_0 is the initial liquid volume; V is the remaining liquid volume; d_0 is the initial distance between top and bottom surfaces of the foldable chamber. Combining equation (1) and (2), the released liquid volume can be represented by a function of capacitance C. The capacitor is electronically connected to the induction coil to form an LC circuit with a resonant frequency f, which is given by

$$f = \frac{1}{2\pi\sqrt{LC}}, \quad (3)$$

Where L is inductance of the induction coil. The resonant frequency can be monitored by the VNA when the foldable chamber has different capacitance values. A calibration is performed to fit a model between the resonant frequency and released liquid volume, which can be later used for *in situ* liquid release volume monitoring.

The quality factor Q of the LC circuit is given by

$$Q = \frac{1}{R}\sqrt{\frac{L}{C}}, \quad (4)$$

where R is the resistance of the LC circuit. The readout distance of the sensing mechanism can be increased by increasing the quality factor. Here, the inductance of the inductor can be estimated as

$$L = \frac{1.1\mu n^2 D_{coil}}{4}, \quad (5)$$

where μ is magnetic permeability; n is the number of turns of the induction coil; D_{coil} is the diameter of the induction coil [1]. As shown in **Fig. S14**, by increasing the number of turns and induction coil diameter, the readout distance of the liquid release volume can be improved.

Lastly, the bladder tissue and the urine it contains may induce a capacitive effect, leading to a shift in the resonance frequency. This shift arises from the additional capacitance incorporated into the LC circuit. The peak frequency can be calibrated when the device is initially placed inside the bladder.

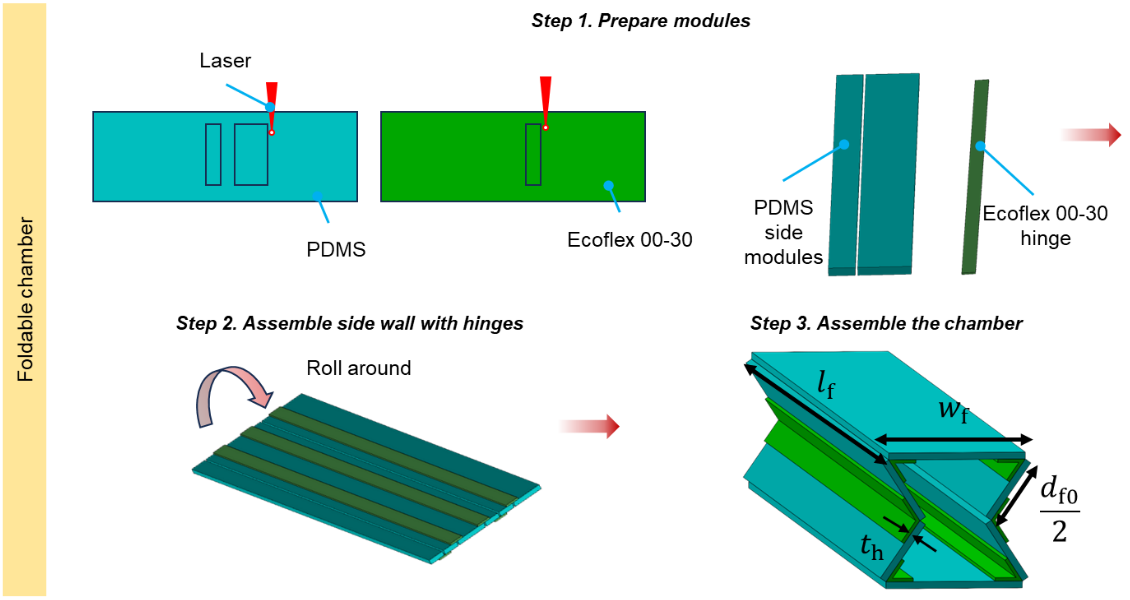

Fig. S1. Fabrication process of the foldable chamber. Step 1: PDMS and Ecoflex 00-30 patches are first prepared. Rectangular PDMS side modules and Ecoflex 00-30 hinges are then cut using a laser cutter. Step 2: The side modules are aligned side by side and connected with the hinges from either the top or bottom surface, depending on the intended folding direction. Step 3: The assembled structure is rolled and bonded along the edges to form a closed foldable chamber. l_{f}, w_{f}, d_{f0} denote the length, width, and initial height of the foldable chamber, respectively, and t_{h} represents the thickness of the Ecoflex 00-30 hinge.

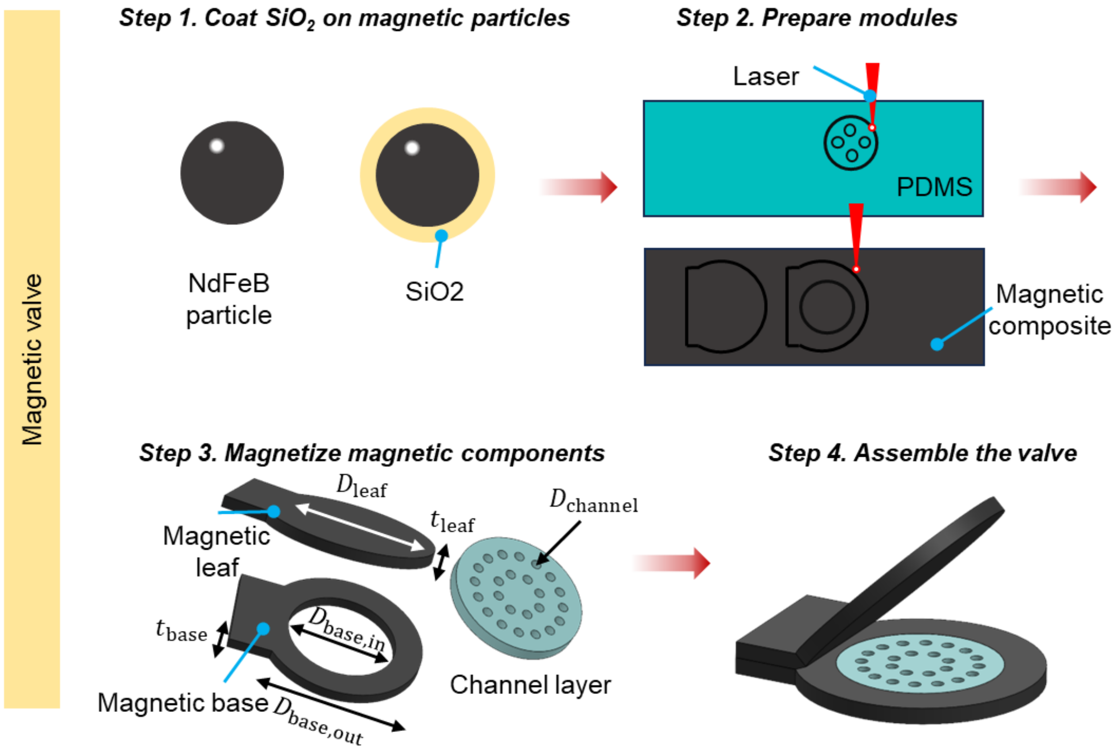

Fig. S2. Fabrication process of the magnetic valve. Step 1: NdFeB microparticles are first coated with SiO_2 to enhance biocompatibility and then used to prepare magnetic composite patches. Step 2: Channel layers are laser-cut from PDMS patches, while the magnetic base and magnetic leaf are cut from the magnetic composite sheets. Step 3: The magnetic leaf and base are magnetized according to the designed magnetization profile. Step 4: The channel layer is embedded within the magnetic base, and the magnetic leaf is bonded to the distal end to form the valve assembly. D_{leaf} and t_{leaf} denote the diameter and thickness of the magnetic leaf, respectively. $D_{\mathrm{base,in}}$, $D_{\mathrm{base,out}}$ and t_{base} represent the inner diameter, outer diameter, and thickness of the magnetic base, respectively. D_{channel} indicates the channel diameter of the PDMS layer.

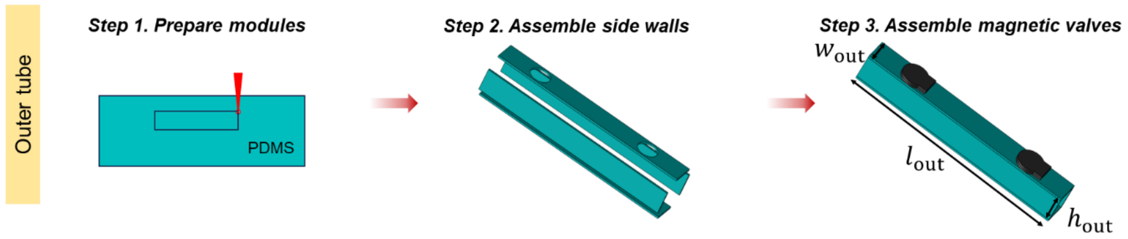

Fig. S3. Fabrication process of the outer tube. Step 1: The sidewall modules of the outer tube are laser-cut from PDMS patches, with one module containing two holes designed for integrating magnetic valves. Step 2: Four sidewall modules are aligned and bonded together to form the tubular structure. Step 3: The magnetic valves are attached to the outer tube, positioned to align precisely with the pre-cut holes on the sidewall. l_{out}, w_{out} and h_{out} denote the length, width, and height of the outer tube, respectively.

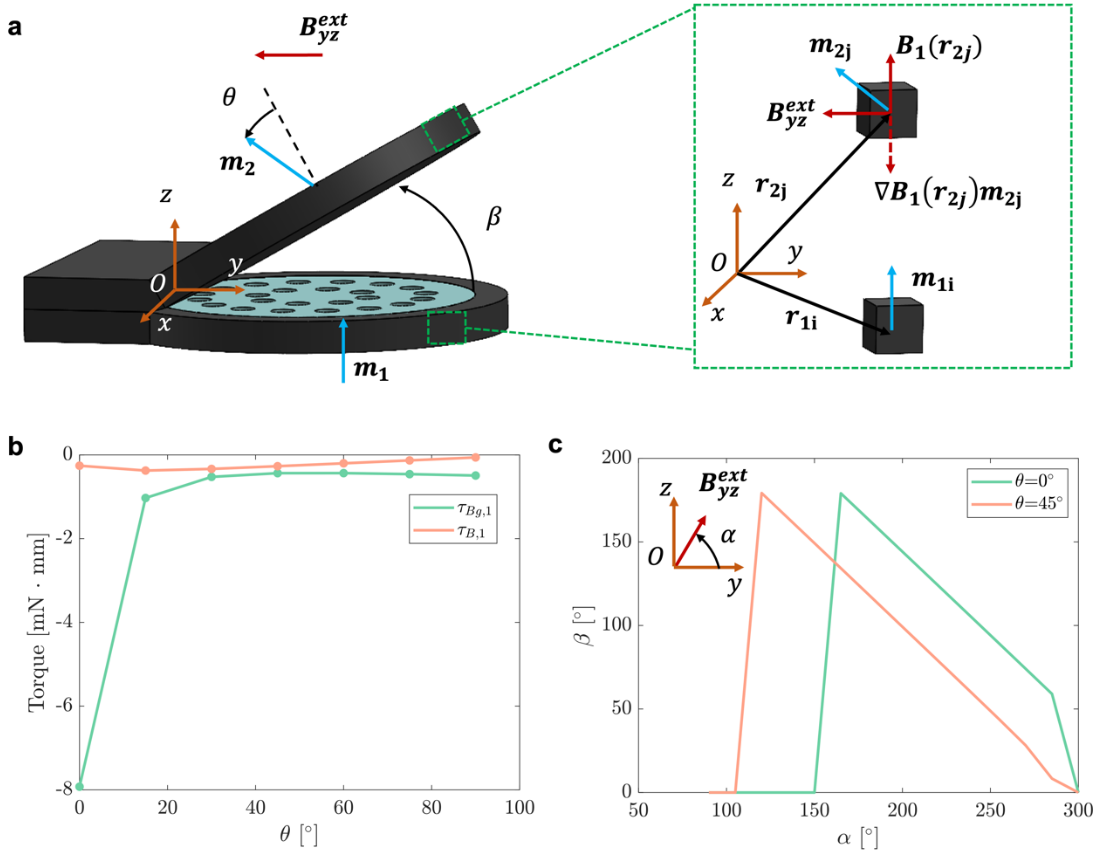

Fig. S4. Schematics of the magnetic valve behavior under external magnetic fields. (a) Illustration of the magnetic valve structure, showing the discretized magnetic leaf and base elements. The coordinate origin OOO is fixed at the midpoint of the rotation axis. (b) Simulated magnetic torques generated by the interaction between the magnetic leaf and base at varying valve opening angles. (c) Comparison of valve opening angles for magnetic valves with different magnetization orientations under a rotating external magnetic field of constant magnitude.

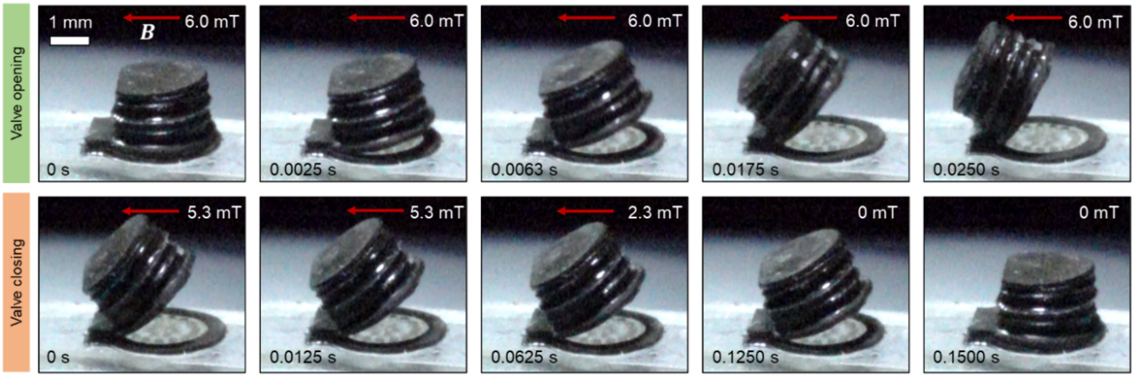

Fig. S5. Sequential high-speed video frames showing the opening and closing process of the magnetic valve. The magnetic leaf has a magnetization of 61.9 kA/m and a thickness of 1.8 mm. Red arrow: magnetic field.

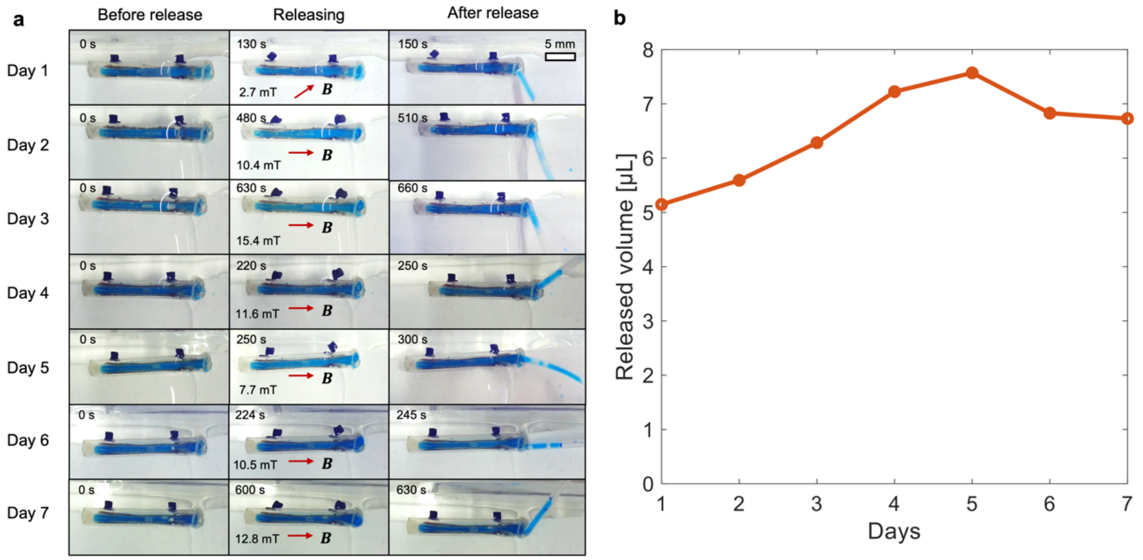

Fig. S6. Seven-day controlled liquid release of the SRP-IDD. (a) Representative video frames of the SRP-IDD before release, during release, and after release in the daily controlled liquid release test. The dispensed liquid was collected through a tube for volumetric measurement. (b) Released liquid volume for each day over the 7-day controlled liquid release experiment.

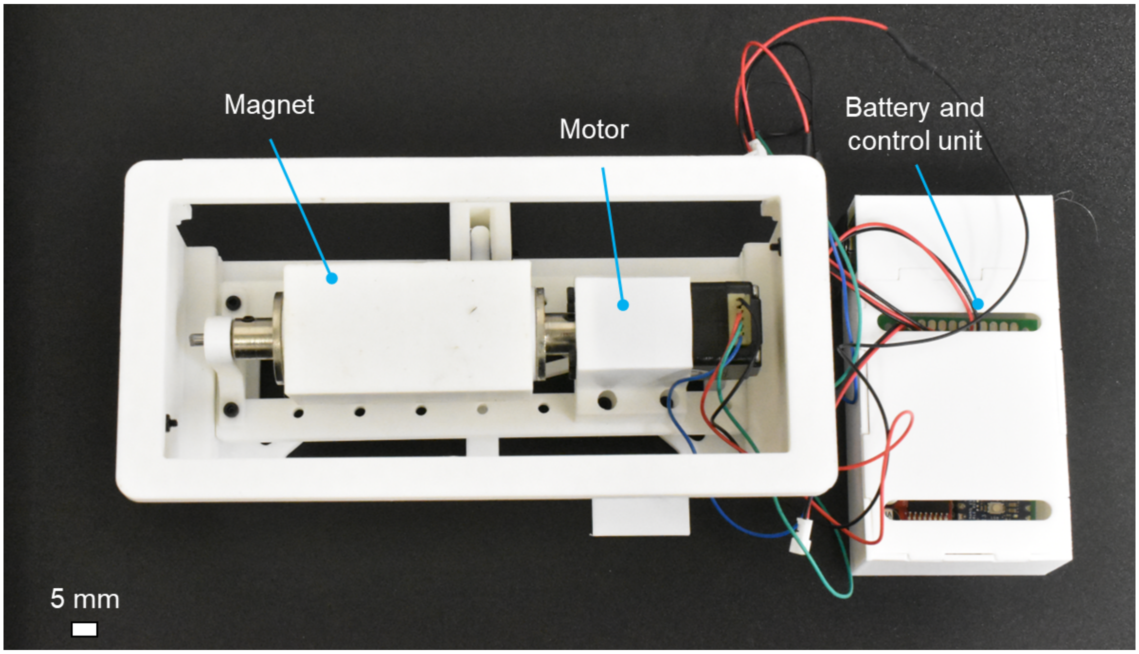

Fig. S7. Optical image of the portable magnetic actuation system. Two permanent magnets are mounted on a stepper motor to generate rotational magnetic fields. The system is powered by a 9 V battery, and motor operation is wirelessly controlled via Bluetooth communication.

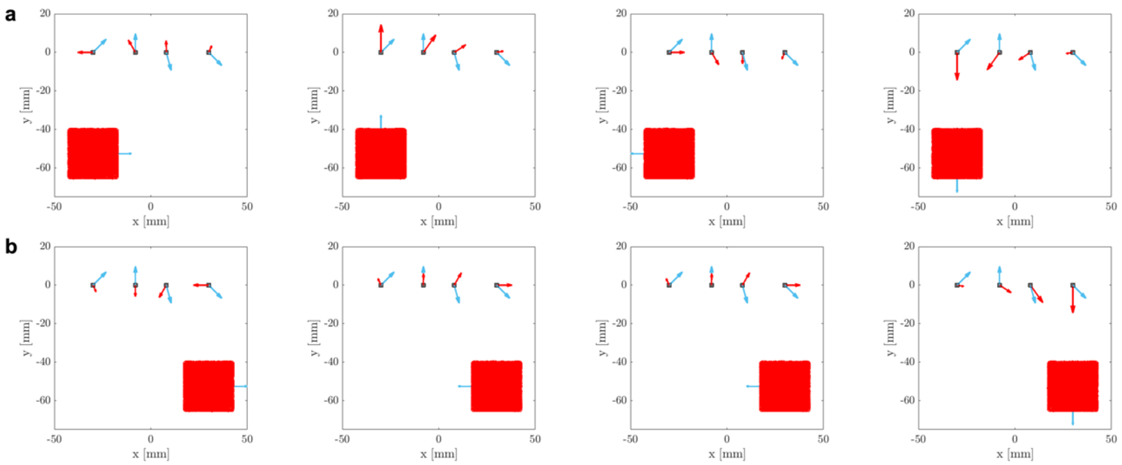

Fig. S8. Magnetic field distribution generated by two cube magnets. (a) Simulated magnetic field distribution of two external actuation magnets positioned with their center at $x = -30$ mm. The red cubes indicate the external magnets, and the black squares mark the centers of the magnetic valves. (b) Magnetic field distribution of the two magnets with the center located at $x = 30$ mm. Each cube magnet measures 25 mm by 25 mm by 25 mm and is composed of NdFeB (grade N42).

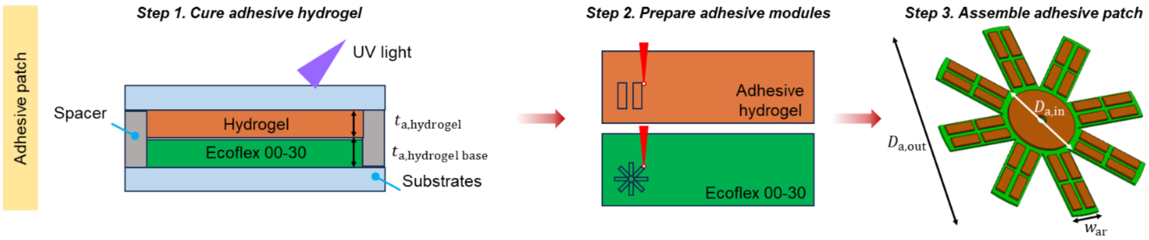

Fig. S9. Fabrication process of the adhesive patch. Step 1: The adhesive hydrogel layer is prepared by curing the hydrogel on an Ecoflex 00-30 base within a mold under UV illumination. Step 2: Adhesive hydrogel modules and the patch base are laser-cut from the cured hydrogel and Ecoflex 00-30 sheets, respectively. Step 3: The hydrogel modules are aligned and bonded onto the base to form the complete adhesive patch. $t_{\text{a,hydrogel}}$ and $t_{\text{a,hydrogel base}}$ base denote the thicknesses of the adhesive hydrogel and base, respectively. $D_{\text{a,out}}$ and $D_{\text{a,in}}$ represent the outer and inner diameters of the adhesive patch, while w_{ar} indicates the width of the adhesive patch arm.

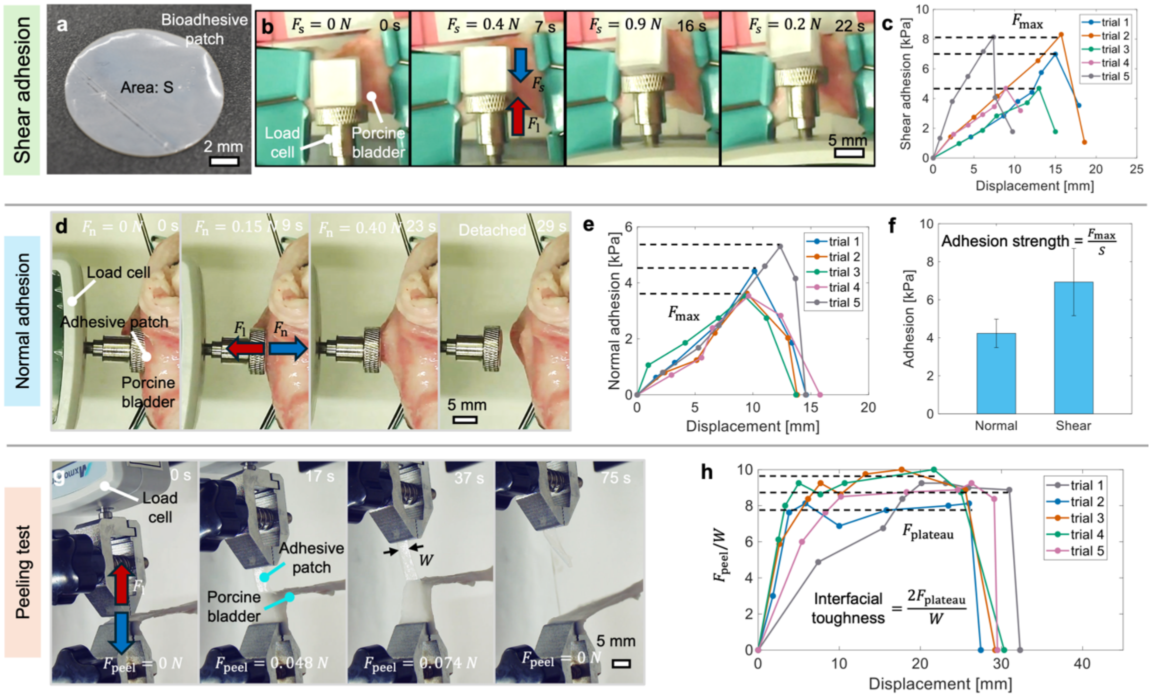

Fig. S10. Quantification of adhesion strength for the bioadhesive. (a) Optical image of the bioadhesive patch used for adhesion strength testing. (b) Sequential video frames of the lap-shear test performed on porcine bladder tissue. (c) Shear adhesion strength as a function of displacement during the lap-shear test. (d) Sequential video frames of the tensile test performed on porcine bladder tissue. (e) Normal adhesion strength as a function of displacement during the tensile test. (f) Average normal and shear adhesion strengths of the bioadhesive. (g) Sequential video frames of the 180° peel test performed on porcine bladder tissue. (h) Adhesion force per unit width as a function of displacement during the 180° peel test. The average interfacial toughness of the bioadhesive is 18.2 N/m. Error bars represent standard deviation ($n = 5$ trials).

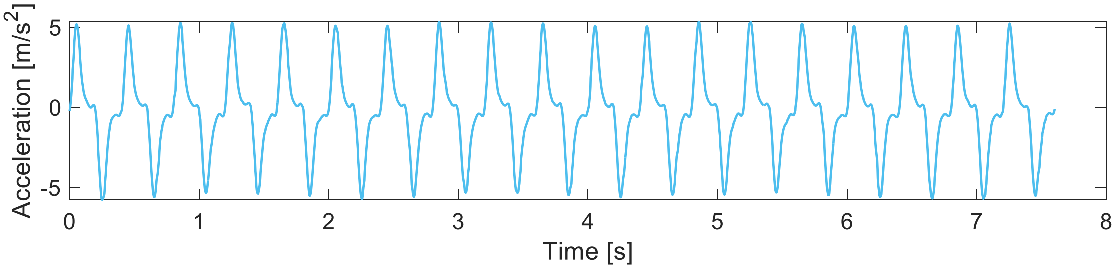

Fig. S11. Measured acceleration of the soft robotic patch on bladder tissues in saline. The data were obtained using an inertial measurement unit (IMU) mounted on the container holding the soft robotic patch during operation.

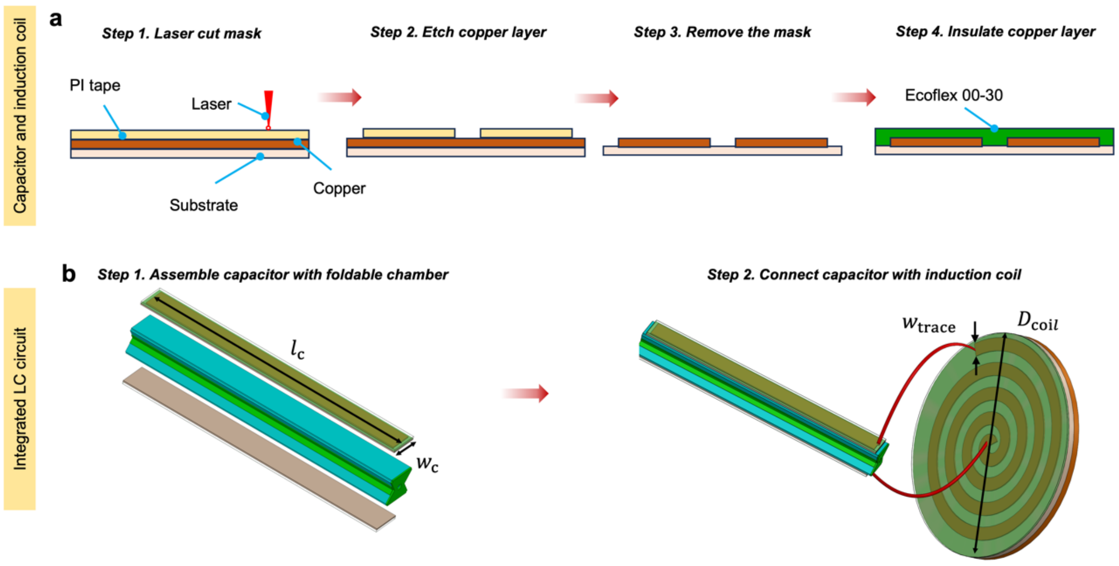

Fig. S12. Fabrication of the capacitor-based sensing unit. (a) Illustration of the capacitor and induction coil fabrication process by etching. Step 1: A patterned mask is first laser-cut. Step 2: The exposed copper layer is etched using a chemical etchant. Step 3: The mask is removed to reveal the patterned copper structure. Step 4: Ecoflex 00-30 is applied as an insulating layer over the copper. (b) Integration of the LC circuit with the foldable chamber. Step 1: The capacitor plates are bonded to the top and bottom surfaces of the foldable chamber. Step 2: The capacitor plates are electrically connected to the induction coil to complete the LC circuit. l_c and w_c denote the length and width of the capacitor plates, w_{trace} represents the width of the induction coil trace, and D_{coil} indicates the diameter of the induction coil.

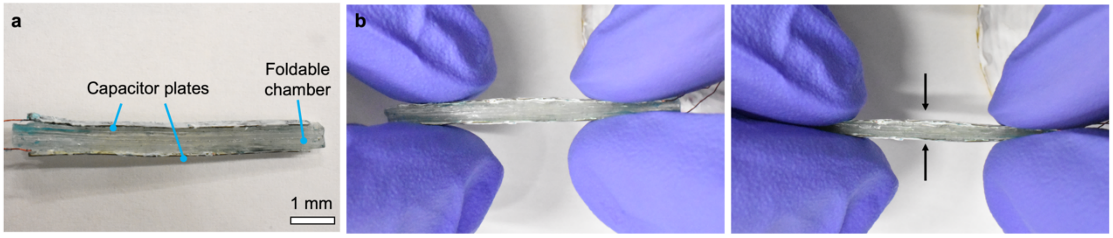

Fig. S13. Optical images of the capacitor-based sensing unit. (a) Optical image showing the capacitor plates integrated with the foldable chamber. (b) Sequential images illustrating the deformation of the foldable chamber with the attached capacitor plates.

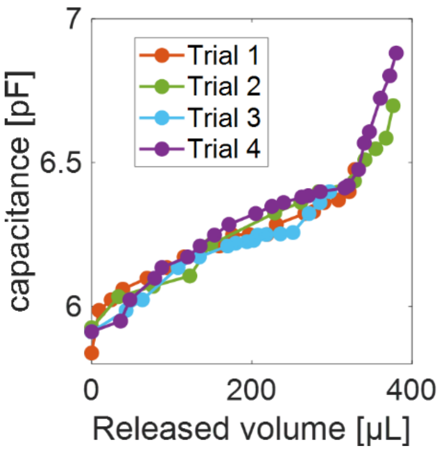

Fig. S14. Capacitance variation as a function of released liquid volume across multiple trials. Four repeated trials were conducted using the same amount of integrated superabsorbent fiber (SAF), showing consistent capacitance–volume response behavior.

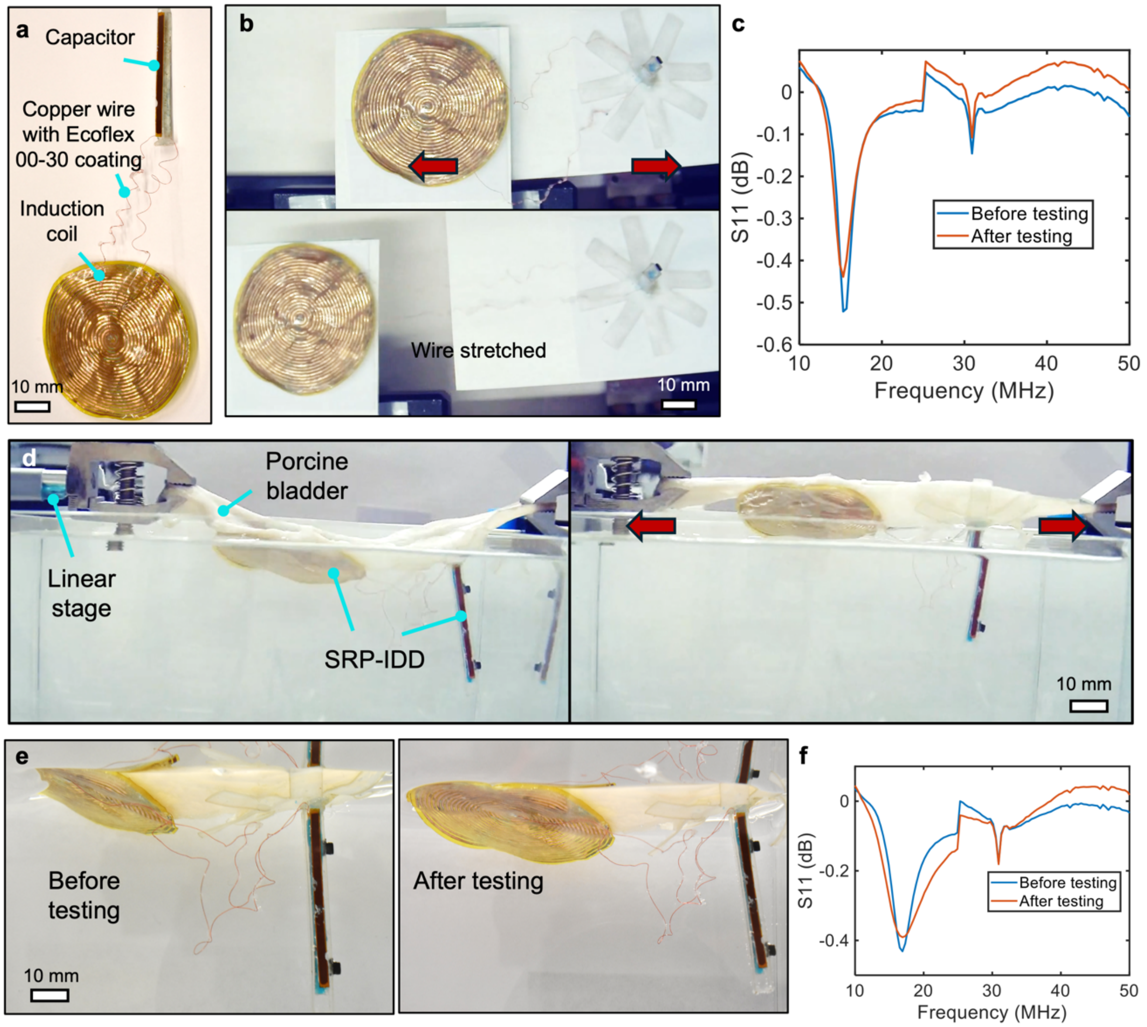

Fig. S15. Fatigue test of the SRP-IDD on *ex vivo* porcine bladder tissue. (a) Optical image of the foldable chamber integrated with the LC circuit. The copper wire connecting the capacitor and induction coil is encapsulated with Ecoflex 00-30. (b) Representative video frames showing the resting and stretched states during the mechanical fatigue test, in which 1000 loading cycles were applied. (c) Measured S_{11} parameter as a function of frequency before and after the mechanical fatigue test. (d) Representative video frames showing the resting and stretched states during the fatigue test with the SRP-IDD attached to a piece of porcine bladder tissue, which was clamped and cyclically stretched using a linear stage for 1000 cycles. (e) Optical images of the SRP-IDD before and after the tissue-mounted fatigue test. (f) Measured S_{11} parameter as a function of frequency before and after the tissue-mounted fatigue test.

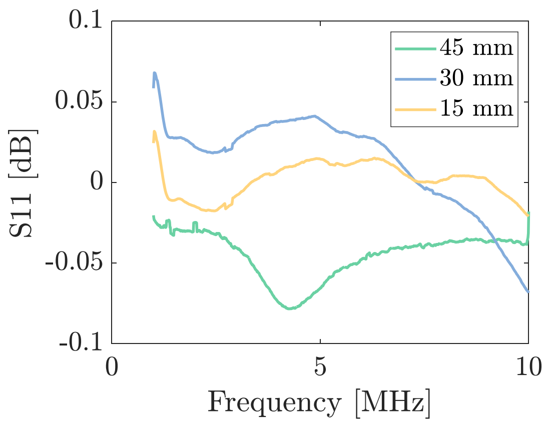

Fig. S16. S_{11} parameter as a function of frequency for induction coils of different diameters on porcine colon tissue in saline. Induction coils with diameters of 15 mm, 30 mm, and 45 mm were integrated with the capacitor. The readout coil, connected to a vector network analyzer (VNA), was positioned 1 cm away from the induction coils during measurement.

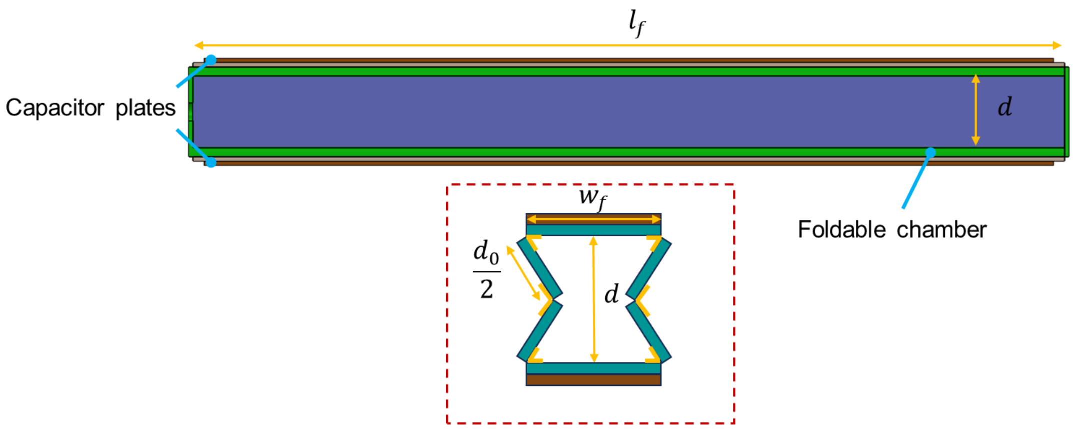

Fig. S17. Illustration of the capacitor integrated with the foldable chamber. Two capacitor plates are attached to the top and bottom surfaces of the foldable chamber. The distance between the plates changes as the chamber deforms, enabling capacitance variation corresponding to the chamber's volume change.

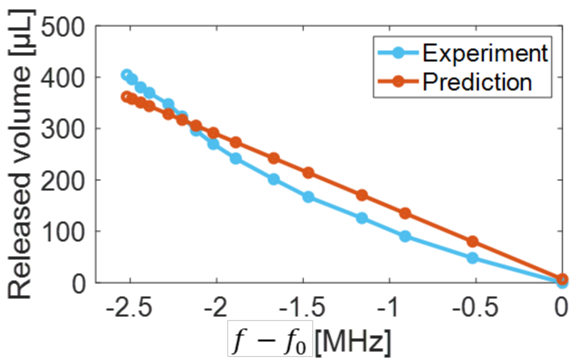

Fig. S18. Comparison between predicted and experimentally measured liquid release volumes. The predicted release volumes were calculated from the detected resonance frequency shifts and show strong agreement with the experimentally measured liquid volumes.

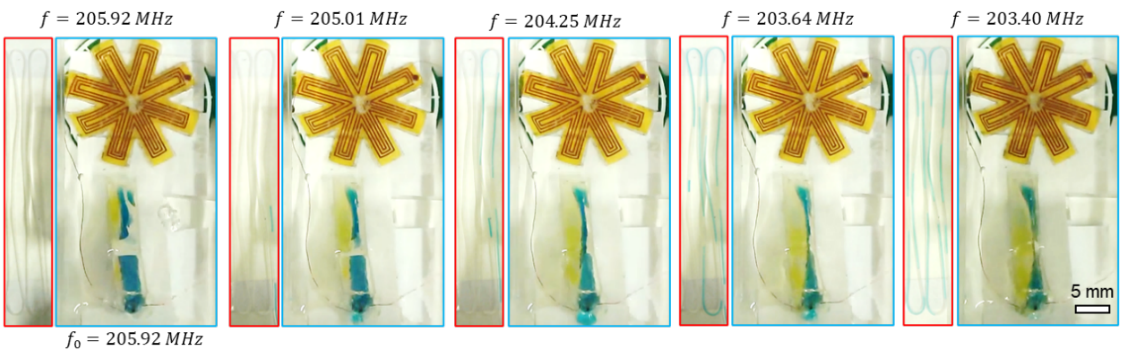

Fig. S19. Sequential video frames showing the liquid release and sensing process. Images within the red boxes show the tubing used to measure released liquid volume as the ground-truth reference. Images within the blue boxes display the induction coil and drug release chamber, with a vector network analyzer (VNA) pickup coil positioned beneath the induction coil for real-time sensing.

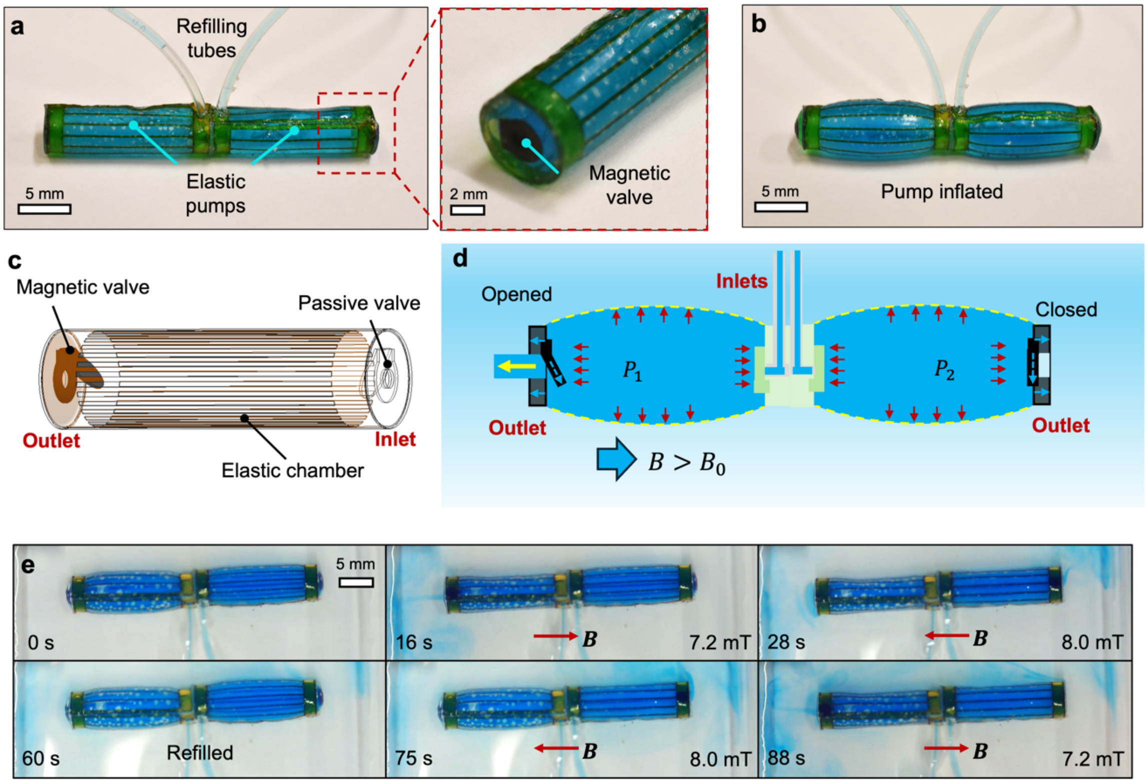

Fig. S20. Elastic pump for multiplexed liquid release with refilling functions. (a) Optical images of refillable elastic pumps with two chambers and magnetic valves integrated. (b) Optical images of refillable elastic pumps in inflated states. (c) Illustration of the elastic pump and the valves. (d) Illustration of multiplexed liquid release. (e) Video frames of sequential release by controlling magnetic valves. After liquids are released from two pumps respectively, additional liquids are filled through the refilling tubes. The refilled liquids are then ejected in opposite sequence.

Table S1 Key parameters and variables

Variables	Definition	Design value
l_{out}	Length of the outer tube	40 mm
w_{out}	Width of the outer tube	2.4 mm
h_{out}	Height of the outer tube	2.4 mm
l_{f}	Length of the foldable chamber	38 mm
w_{f}	Width of the foldable chamber	2.0 mm
d_{f0}	Initial height of the foldable chamber	2.0 mm
t_{h}	Thickness of the foldable chamber hinge	50 μm
l_{c}	Length of the capacitor	36 mm
w_{c}	Width of the capacitor	2.0 mm
D_{coil}	Diameter of the induction coil	45 mm
w_{trace}	Width of the induction coil trace	0.8 mm
n_{coil}	Number of turns of the induction coil	22
D_{channel}	Diameter of the channel	150 μm
$D_{\mathrm{base,in}}$	Inner diameter of the magnetic base	2.0 mm
$D_{\mathrm{base,out}}$	Outer diameter of the magnetic base	2.4 mm
D_{leaf}	Diameter of the magnetic leaf	2.5 mm
t_{leaf}	Thickness of the magnetic leaf	1.8 mm
t_{base}	Thickness of the magnetic ring-shaped base	0.2 mm
$D_{\mathrm{a,out}}$	Outer diameter of the adhesive patch	45 mm
$D_{\mathrm{a,in}}$	Inner diameter of the adhesive patch	15 mm
w_{ar}	Width of the adhesive patch arm	6 mm
$t_{\mathrm{a,hydrogel}}$	Thickness of the adhesive hydrogel	50 μm
$t_{\mathrm{a,hydrogel\ base}}$	Thickness of the adhesive hydrogel base	200 μm

SI videos.

Movie S1. Mechanism of pump and sensing for intravesical liquid release.
This video demonstrates the pumping mechanism based on the superabsorbent fiber (SAF) and the sensing capability of liquid release using a capacitor-based folding mechanism.

Movie S2. Coordinated control of valves for regulated liquid release.
This video shows liquid release control using a single magnetic valve, followed by coordinated actuation of multiple flexible magnetic valves for multiplexed regulation.

Movie S3. Retention of the device on porcine bladder tissues.
This video demonstrates the retention performance of the SRP-IDD on porcine bladder tissues under physiological conditions.

Movie S4. Delivery of the medical patch on porcine bladder tissues.
This video illustrates the delivery process of the SRP-IDD onto porcine bladder tissues using a cystoscope.

Movie S5. On-demand and controlled liquid release with sensing.
This video shows on-demand, controlled liquid release and real-time sensing of the SRP-IDD on porcine bladder tissues.

References

[1] Mohan, S.S., del Mar Hershenson, M., Boyd, S.P. and Lee, T.H., 2002. Simple accurate expressions for planar spiral inductances. *IEEE Journal of solid-state circuits*, *34*(10), pp.1419-1424.
